# LMI1 homeodomain protein regulates organ proportions by spatial modulation of endoreduplication

**DOI:** 10.1101/gad.318212.118

**Published:** 2018-11-01

**Authors:** Francesco Vuolo, Daniel Kierzkowski, Adam Runions, Mohsen Hajheidari, Remco A. Mentink, Mainak Das Gupta, Zhongjuan Zhang, Daniela Vlad, Yi Wang, Ales Pecinka, Xiangchao Gan, Angela Hay, Peter Huijser, Miltos Tsiantis

**Affiliations:** 1 Deparment of Comparative Development and Genetics, Max Planck Institute for Plant Breeding Research, 50829 Cologne, Germany;; 2 Department of Plant Sciences, University of Oxford, Oxford OX1 3RB, United Kingdom;; 3 Department of Plant Breeding Genetics, Max Planck Institute for Plant Breeding Research, 50829 Cologne, Germany

**Keywords:** plant homeobox, leaf development, organ proportions, live imaging

## Abstract

How the interplay between cell- and tissue-level processes produces correctly proportioned organs is a key problem in biology. In plants, the relative size of leaves compared with their lateral appendages, called stipules, varies tremendously throughout development and evolution, yet relevant mechanisms remain unknown. Here we use genetics, live imaging, and modeling to show that in *Arabidopsis* leaves*,* the LATE MERISTEM IDENTITY1 (LMI1) homeodomain protein regulates stipule proportions via an endoreduplication-dependent trade-off that limits tissue size despite increasing cell growth. LM1 acts through directly activating the conserved mitosis blocker *WEE1*, which is sufficient to bypass the *LMI1* requirement for leaf proportionality.

## Abstract (precis)

Here, Vuolo et al. investigated the mechanisms controlling the relative size of leaves compared with their lateral appendages (stipules). Using genetics, live imaging, and modeling, they demonstrate that the LATE MERISTEM IDENTITY1 (LMI1) homeodomain protein regulates stipule proportions via an endoreduplication-dependent trade-off that limits tissue size despite increasing cell growth.

How spatiotemporal coordination of cell and tissue growth contributes to plant and animal form is a key question in biology. Plant leaves are a powerful system to study growth and form because they show complex and diverse geometries that can be studied genetically. Leaf growth typically involves a phase of cell proliferation early in development followed by cell expansion associated with endoreduplication (Melaragno et al. 1993). However, developmental inputs into endoreduplication and how these shape leaf form remain largely unknown (Walker et al. 2000; Cookson et al. 2005; Massonnet et al. 2011). A key feature of leaf shape is the production of repeated marginal protrusions. These outgrowths vary from slight serrations in simple leaves to distinct leaflets in dissected leaves (Hay and Tsiantis 2006; Bilsborough et al. 2011; Hasson et al. 2011; Bar and Ori 2014; Vlad et al. 2014; Rast-Somssich et al. 2015). Stipules are another type of outgrowth that contribute considerably to macroevolutionary diversity in leaf form (Sinnott and Bailey 1914), a possibility also introduced by Darwin (1865); however, the mechanisms that influence stipule growth and development are poorly understood. Stipules typically flank the leaf base and vary in morphology from vestigial structures, as in *Arabidopsis thaliana*, to large leafy photosynthetic units, as in the pea. In *A. thaliana*, stipules initially comprise a significant proportion of the leaf primordium but only a small fraction of the mature leaf length (Fig. 1A–C). This indicates strong allometric regulation of leaf versus stipule growth, the genetic basis of which is unknown.

**Figure 1.**
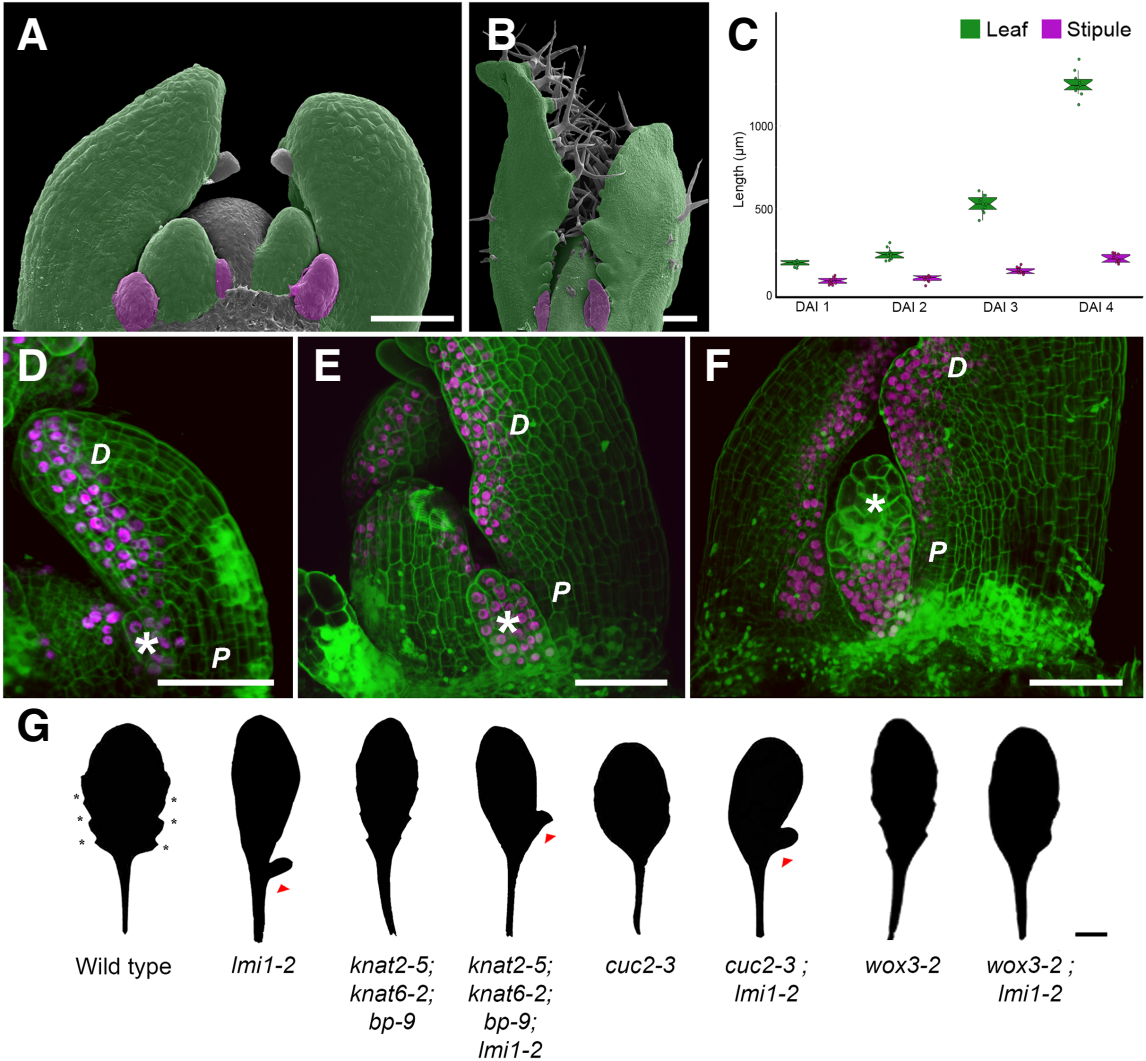
*LATE MERISTEM IDENTITY1* (*LMI1*) is expressed in the distal leaf domain and stipule. (*A*–*C*) Wild-type leaves (green) and stipules (purple) in false-colored scanning electron micrographs at 2 d after leaf initiation (DAI) (*A*) and 8 DAI (*B*) and length (in micrometers) at successive DAI (*C*). *n* = 10 leaves. Mean ± SEM. (*D*–*F*) Confocal laser scanning microscopy (CLSM) of *LMI1::LMI1:VENUS* expression (magenta) in propidium iodide (PI)-stained (green) leaf primordia at 3 DAI (*D*), 5 DAI (*E*), and 7 DAI (*F*). *n* = 5 independent T2 lines. (Asterisks) Stipules; (D) distal leaf domains; (P) proximal leaf domains. Bars: *A*,*B*,*D*–*F*, 50 µm. (*G*) Silhouettes of adult leaves from wild-type and mutant plants. For penetrance scoring, see Supplemental Table 1. (Triangles) Ectopic lobes; (asterisks) serrations. Bar, 1 cm.

## Results and Discussion

To identify molecular mechanisms required to yield correctly proportioned leaves, we investigated the HD-ZIP class I transcription factor LATE MERISTEM IDENTITY1 (LMI1) (Saddic et al. 2006), which regulates leaf growth in *A. thaliana* (Vlad et al. 2014)*. LMI1* expresses in the distal leaf margin (Fig. 1D–F), where serrations fail to form in *lmi1* mutant leaves (Fig. 1G; Supplemental Fig. 1A–F; Saddic et al. 2006; Vlad et al. 2014; Vuolo et al. 2016). Conversely, *LMI1* is not expressed in the proximal leaf margin (Fig. 1D–F), yet *lmi1* leaves display ectopic lobes in this region of the leaf (Fig. 1G; Supplemental Fig. 1C,D,G–K; Saddic et al. 2006). The smooth distal margin of *lmi1* leaves is consistent with LMI1 acting as a growth repressor (Vlad et al. 2014); for example, mutations in the local growth repressors *REDUCED COMPLEXITY* (*RCO*) and *CUP-SHAPED COTYLEDON 2* (*CUC2*) lead to smoother leaves (Bilsborough et al. 2011; Vlad et al. 2014). However, the lobed proximal margin of *lmi1* leaves is difficult to reconcile with LMI1 function. Surprisingly, lobe formation in *lmi1* does not require *CUC2* or *KNOTTED-LIKE HOMEOBOX* (*KNOX*) gene function, which are known regulators of lobe development (Fig. 1G; Supplemental Fig. 1K; Lincoln et al. 1994; Bilsborough et al. 2011; Hasson et al. 2011; Rast-Somssich et al. 2015). *LMI1* expresses in developing stipules (Fig. 1D,E), eventually becoming restricted to the proximal part of mature stipules (Fig. 1F). Therefore, we hypothesized that LMI1 may act in stipules to limit their growth, leading to their excess growth into lobes in the *lmi1* mutant. In this case, genetic ablation of stipules should remove ectopic lobes in *lmi1* leaves. To test this idea, we used a stipule-less mutant of *WUSCHEL-RELATED HOMEOBOX 3* (*WOX3*) (Shimizu et al. 2009) and found no lobed margins in *wox3-2;lmi1-2* double mutants (Fig. 1G), strongly suggesting that the lobed margin in *lmi1* leaves results from a transformation of the stipule into a leaf.

**Figure 2.**
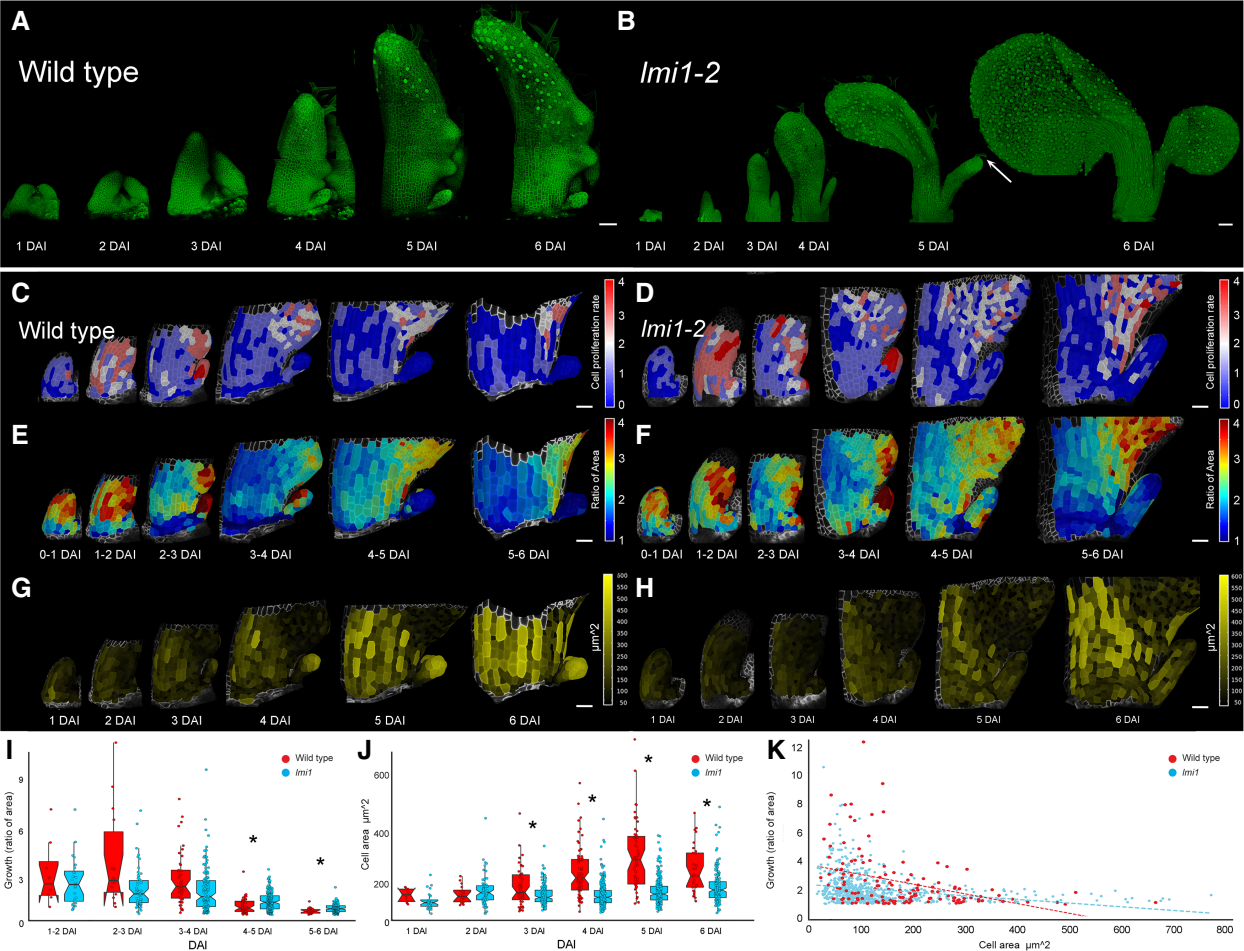
*LMI1* represses tissue growth and cell proliferation in stipules. (*A*,*B*) Time-lapse CLSM of leaf 11 developing over 1–6 DAI in wild type (*A*) and *lmi1-2* (*B*). Cells are outlined by PM-YFP expression. (Arrow) Trichome. (*C*–*H*) Cell proliferation rate (number of divisions; *C*,*D*), growth (ratio of areas; *E*,*F*), and cell area (*G*,*H*) quantified in wild-type (*C*,*E*,*G*) and *lmi1-2* (*D*,*F*,*H*) leaf 11 time-lapse series. Scales are shown in heat maps. *n* = 5. (*I*,*J*) Quantification of cell growth (ratio of areas; *I*) and area (square micrometers; *J*) in wild-type (red) and *lmi1-2* (blue) leaf 11. *n* = 3 time-lapse series; *n* > 50 cells. Mean ± SD. (*) *P* < 0.05, KS-test (*I*) and Student's *t*-test (*J*). (*K*) Cell area and growth values pooled for all DAI in wild-type (red; *n* = 139 cells) and *lmi1-2* (blue; *n* = 690 cells) leaf 11. Dashed lines represent linear regressions. Bars: *A*–*H*, 50 µm.

To verify whether basal lobes of *lmi1* mutants are overgrown stipules and investigate the cellular basis of this transformation, we performed time-lapse imaging of growing leaves (Fig. 2A–H; Barbier de Reuille et al. 2015). Wild-type stipules show high rates of cell proliferation and growth starting 1 d after leaf initiation (DAI) (Fig. 2C,E,I). By 3 DAI, cell proliferation decreased dramatically, as did cell growth at 4 DAI (Fig. 2C,E,I). In contrast, cell proliferation and growth were maintained for longer in *lmi1* stipules, with cells still dividing at 5–6 DAI (Fig. 2D), leading to smaller cells in *lmi1* than wild-type stipules (Fig. 2G,H,J). In addition, clonal sectors derived from lineage tracing analysis were considerably more elongated in *lmi1* stipules (Supplemental Fig. 2A–D). Thus, growth is higher in *lmi1* than wild-type stipules from 3 DAI onward and proceeds for longer (Fig. 2E,F,I). Growth rate and cell area correlate negatively in these tissues, indicating that tissue growth reduces as cells enlarge (Fig. 2K). This correlation was weaker for *lmi1* than wild-type stipules, suggesting that its underlying mechanism requires *LMI1* (Fig. 2K).

Although *LMI1* is expressed in at least two outer cell layers, stipules originate from epidermal founder cells (approximately one to five cells) (Supplemental Figs. 2A,C,E–G, 3A–C). In contrast, *lmi1* stipules initiate from a larger number of founder cells (approximately seven to 12 cells epidermally) (Supplemental Fig. 3D–F) that can also include cells from internal layers (Supplemental Fig. 2H,I). This suggests that *LMI1* represses stipule size at least in part by restricting stipule initial cells to the epidermis and limiting their number. We also observed stomata and trichome cells in *lmi1* stipules, normally present only in the leaf and not in wild-type stipules (Fig. 2A,B). Therefore, in the absence of *LMI1*, more stipule initial cells are recruited to form a larger leaf-like outgrowth. In wild type, the stipule base is very narrow and stays attached to the boundary zone between the leaf base and adjacent tissue (Supplemental Fig. 2C). In contrast, the *lmi1* stipule base grows together with adjacent petiole cells, progressively fusing the stipule with the leaf (Supplemental Figs. 1A–D,G–J, 2C,D). The extent of this fusion is variable, however, and the presence or absence of a lobe results from incomplete or complete fusion, respectively. The transformed stipules are more distally located (Fig. 1G) than their wild-type counterpart present at the leaf base, which is consistent with them initiating in a faster-growing context (Fig. 2E,F). *lmi1* stipules also grow anisotropically, more akin to the leaf petiole than to wild-type stipules (Supplemental Fig. 2J–M). The length of *lmi1* stipules reaches three times the length of wild-type stipules (Supplemental Fig. 4A,B), thus altering the allometric proportions of stipule to leaf in *lmi1* leaves (Supplemental Fig. 4C,D). Overall, our findings suggest that *LMI1* restricts stipule size by limiting founder cell recruitment and advancing cells from proliferative to expansive growth such that cell size increases but tissue growth is reduced.

**Figure 3.**
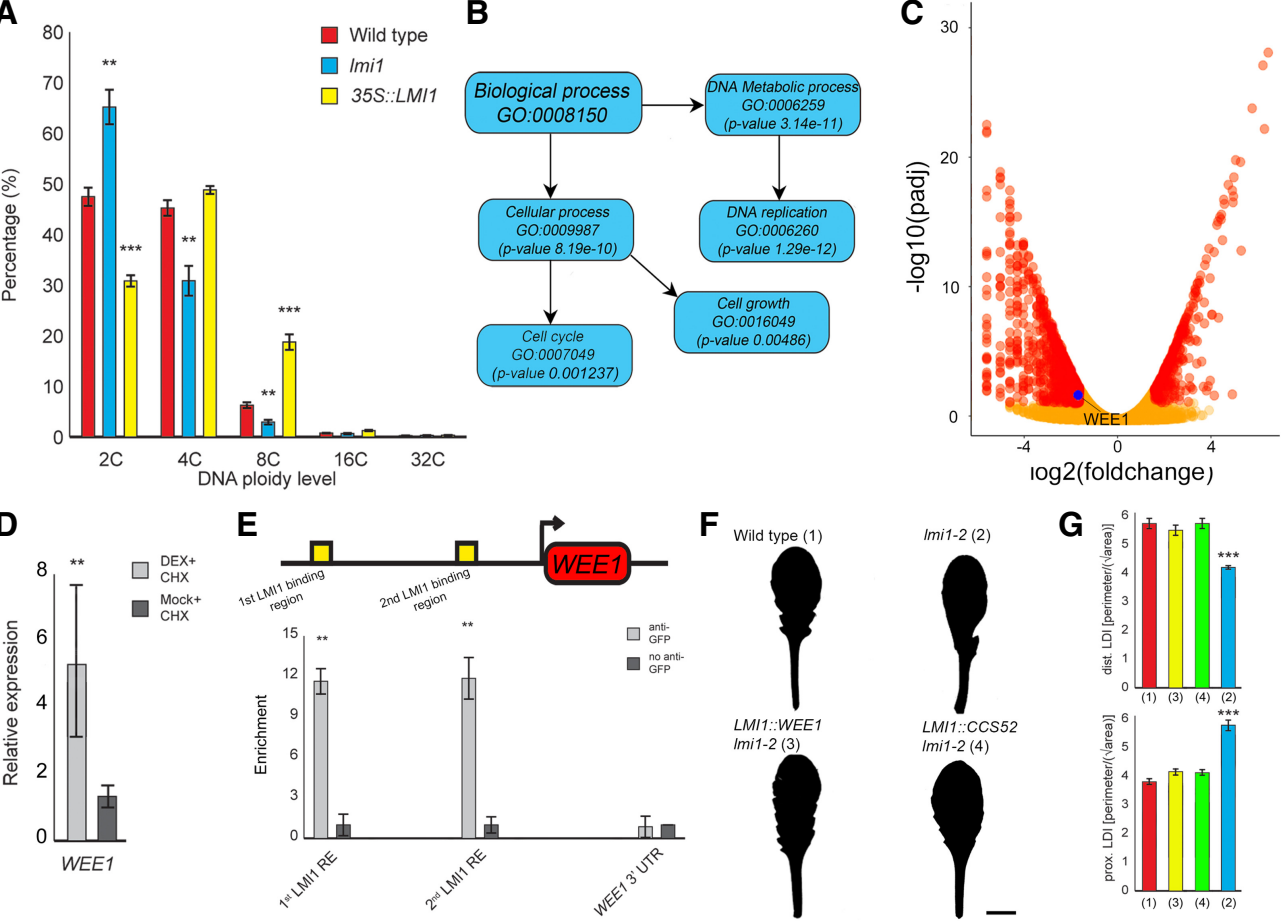
*LMI1* promotes endoreduplication by activating *WEE1* expression. (*A*) Ploidy analysis by flow cytometry in wild-type (red), *lmi1-2* (blue), and *35S::LMI1* (yellow) leaves. *n* = 5. Mean percentage ± SEM. (**) *P* < 0.01; (***) *P* < 0.001, ANOVA. (*B*) Subgroup of the GO categories enriched among differentially expressed genes (DEGs) between wild type and *lmi1-2*, derived from RNA-seq analysis. *n* = 3. (*C*) Volcano plot showing DEGs (red) and non-DEGs (orange) in *lmi1-2* compared with wild-type seedlings, and the *WEE1* gene with a putative LMI1-binding site (blue). (*D*) Quantitative RT–PCR (qRT–PCR) of *WEE1* expression in *lmi1-2;LMI1::LMI1:GR* plants treated with dexamethasone (DEX) + cycloheximide (CHX) (gray) or mock + CHX (dark gray) 3 h after treatment. *n* = 3. Mean ± SEM. (**) *P* < 0.01, Student's *t*-test. (*E*, *top*) *WEE1* gene model with upstream regions containing putative LMI1-binding sequences marked in yellow. The arrow indicates transcription start. (*Bottom*) ChIP-qRT–PCR (chromatin immunoprecipitation [ChIP] combined with qRT–PCR) after anti-GFP (gray) or control IgG (dark gray) pull-down in *LMI1::LMI1:VENUS* shows significant association of LMI1 with chromatin in regions containing the putative binding sites. *n* = 3. Mean ± SEM. (**) *P* < 0.0, Student's *t*-test1. *WEE1* 3′ untranslated region (UTR) was used as negative control. (*F*) Silhouettes of leaf 11 in wild-type, *lmi1-2*, *lmi1-2;LMI1::WEE1,* and *lmi1-2;LMI1::CCS52* plants. *n* = 15 T2 lines. (*G*) Dissection indices of the distal domain (*top* graph) and proximal domain (*bottom* graph) in the lines in *F*, with relative numbers matching the ones in *F*. *n* = 10, each line. Bar, 1 cm.

Endoreduplication counters cell proliferation, promotes cell enlargement, and can influence cell identity (Szymanski and Marks 1998; Bramsiepe et al. 2010; Maruyama et al. 2011; Roeder et al. 2012); therefore, we hypothesized that *LMI1* might affect cell division, cell size, and organ identity in the stipule by promoting endoreduplication. Comparing DNA ploidy levels in wild-type, *lmi1*, and broadly expressing *35S::LMI1* plants (Fig. 3A), we found that the *lmi1* mutant has 27% more 2C nuclei but 35% less 4C nuclei and 50% less 8C nuclei compared with wild type. In contrast, the *35S::LMI1* transgenic line shows almost 150% more 8C nuclei and 30% less 2C nuclei than wild-type samples. These observations indicate that *LMI1* is necessary and sufficient to define the wild-type leaf endoreduplication profile (Fig. 3A). Consistent with these findings, leaf trichomes were excessively branched in *35S::LMI1* compared with wild type (Supplemental Fig. 5), a phenotype linked to increased endoreduplication (Walker et al. 2000). Additionally, we found that cell size and polytene regions (fused sister chromatids that form after endoreduplication) were reduced in the stipules and leaf margin of *lmi1* (Supplemental Fig. 6), further indicating that *LMI1* promotes endoreduplication in the leaf base. To explore cellular processes influenced by *LMI1* at the whole-genome level, we used RNA sequencing (RNA-seq) to compare wild-type and *lmi1* seedling transcriptomes. We found that differentially expressed genes were enriched for gene ontology (GO) terms related to the cell cycle, cell growth, and DNA replication (Fig. 3B), consistent with *LMI1* promoting endoreduplication.

**Figure 4.**
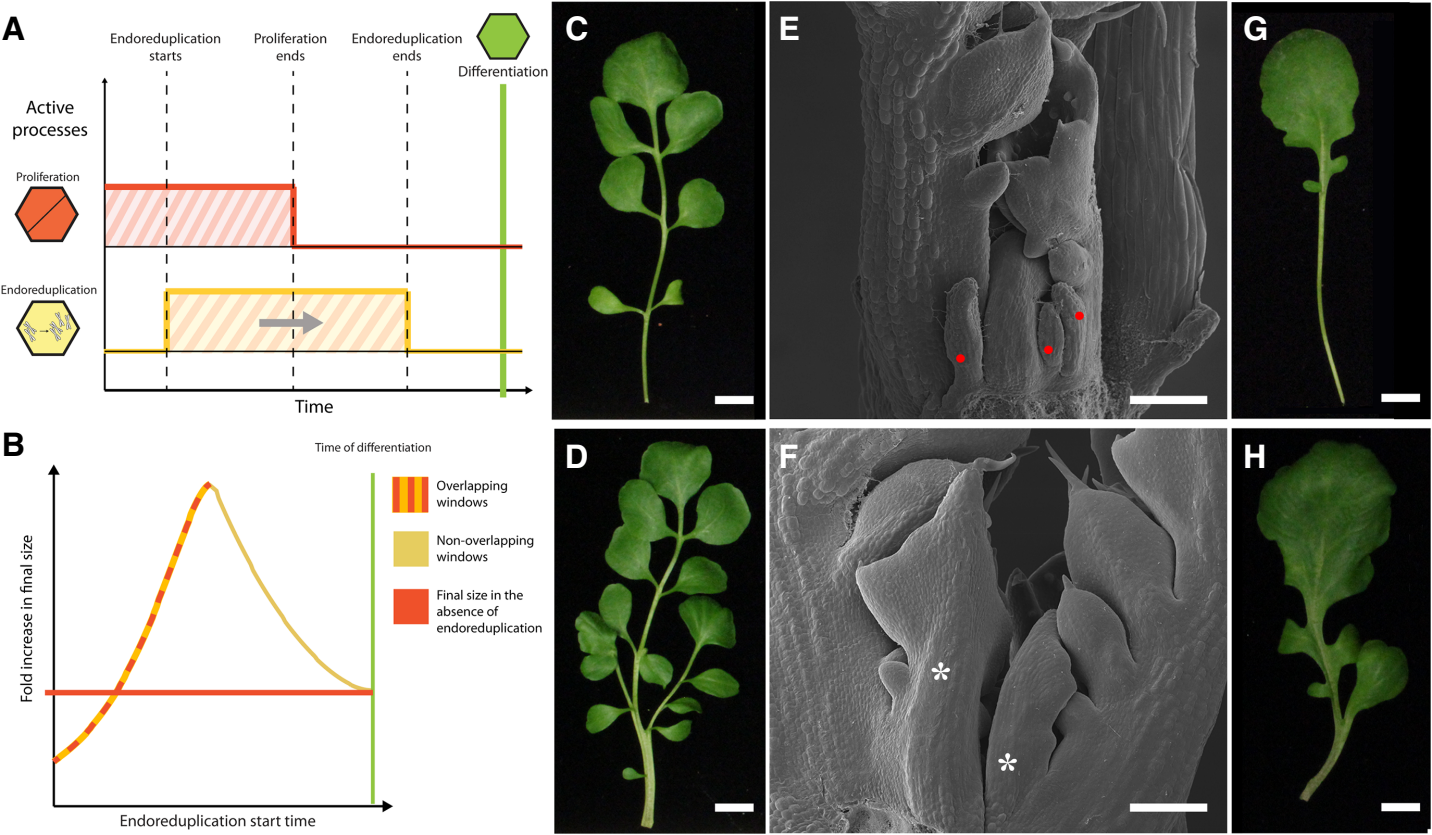
Modeling the regulation of organ size by endoreduplication and conservation of *LMI1* function in *Cardamine hirsuta*. (*A*,*B*) Cell population model incorporating proliferation, endoreduplication, and differentiation. (*A*) The start time for endoreduplication (yellow line) is varied relative to the window of proliferation (orange line), and both processes are terminated by differentiation (green line). (*B*) Simulations showing fold increase of organ size as a function of endoreduplication start time relative to the size increase produced by proliferation alone (orange line; i.e., when *R*_*e*_ = 0). The color of the curve indicates when windows of proliferation and endoreduplication overlap in time (yellow and orange) or occur sequentially (yellow only). (*C–H*) Leaves of wild-type (*C*,*E*), *35S::amiR-LMI1* (*D*,*F*), *rco* (*G*), and *35S::amiR-LMI1*;*rco* (*H*) representative leaf 5 shown for *n* = 15 independent lines per genotype. Bars: *C*,*D*,*G*,*H*, 1 cm; *E*,*F* (scanning electron micrographs of developing leaves), 100 µm. (Red dots) Stipules; (asterisks) ectopic leaves.

One of the key cell cycle genes showing reduced expression in *lmi1* is *WEE1* (Fig. 3C), which can inhibit mitosis, may promote endoreduplication (De Schutter et al. 2007; Gonzalez et al. 2007; Chevalier et al. 2011), and is expressed in both leaves and stipules (Supplemental Fig. 7). *WEE1* is likely to be a direct target of LMI1, as it is transcriptionally up-regulated upon treatment with dexamethasone and the protein synthesis inhibitor cycloheximide in plants harboring a glucocorticoid-inducible *LMI1* transgene (*LMI1::LMI1-GR*) (Fig. 3D). Consistent with this idea, we amplified *WEE1* regulatory regions from chromatin immunoprecipitated using anti-GFP and found enrichment of LMI1:VENUS at two regions with predicted LMI1-binding sites (CAATwAT, where w is A or T) (Fig. 3E; Franco-Zorrilla et al. 2014). To determine whether *WEE1* is critical for *LMI1* function, we expressed *LMI1::WEE1* in *lmi1* mutants, which restored wild-type leaf shape, indicating that *WEE1* expression suffices to bypass the requirement for LMI1 in leaf development (Fig. 3F,G). Although *wee1* mutant leaves resemble wild type (De Schutter et al. 2007), this background was sufficient to ameliorate the growth repression caused by *35S::LMI1* (Vlad et al. 2014), underscoring the importance of WEE1 for LMI1 function (Supplemental Fig. 8). These findings suggest that *LMI1*-dependent endoreduplication regulates leaf form by locally restricting cell proliferation. To further test this idea, we expressed *LMI1::CCS52*, a known regulator of endoreduplication that is *LMI1*-independent (Fig. 3F; Cebolla et al. 1999), and found it sufficient to rescue both the lobed leaf and serration phenotype of *lmi1* mutants (Fig. 3F,G). In addition, *35S::LMI1* plants developed smaller leaves (Supplemental Figs. 5A, 8D; Vlad et al. 2014). These results indicate that activating endoreduplication may limit final organ size. However, these findings are in contrast to previous reports showing that endoreduplication is associated with increased organ size in fruits and leaves (Melaragno et al. 1993; Gonzalez et al. 2007; Chevalier et al. 2011; Massonnet et al. 2011). We hypothesized that the interplay between cell proliferation and endoreduplication and the relative timing of their activation may be critical to determine final organ size. To formally examine this possibility, we constructed a minimal cell population model (Fig. 4A,B; Supplemental Fig. 9A,B; see the Supplemental Material for details). This model relates organ size to the timing of proliferation and endoreduplication within a finite window preceding differentiation. The model shows that endoreduplication leads to an increase in organ size except when activated very early. In this case, the decrease in cell number cannot be compensated for by the increase in cell size resulting from endoreduplication (Fig. 4B; Supplemental Fig. 9C,D). These results show that the timing of cells switching from proliferation to endoreduplication is critical and that early activation of endoreduplication may significantly reduce organ size.

By exploring the model's parameter space, we found that the start time of endoreduplication should have the largest effect on organ size in the context of highly proliferative tissues (i.e., when *R*_*S*_ is large) (Supplemental Fig. 9D). In proliferative tissues, endoreduplication reduces cell division and, ultimately, cell number. For highly proliferative tissues, this loss exceeds what can be compensated for by the subsequent endoreduplication-driven increase in cell size. Therefore, the exclusion of *LMI1* from the proximal leaf margin (Fig. 1E,F), a highly proliferative region (Kuchen et al. 2012; Vlad et al. 2014), should be crucial to maintain the correct size and shape of *A. thaliana* leaves. Specifically, the model predicts a reduction in final leaf size if endoreduplication is activated very early in this region of the leaf margin. To directly test this prediction, we fused *LMI1* to three different promoters that express both earlier and more proximally than *LMI1* in the leaf margin (*RCO* and *CUC2*) and throughout the leaf primordium (*ASYMMETRIC LEAVES1* [*AS1*]) (Bilsborough et al. 2011; Hasson et al. 2011; Vlad et al. 2014). Leaf size was dramatically reduced in *RCO::LMI1* (Vuolo et al. 2016), *CUC2::LMI1*, and *AS1::LMI1* plants (Supplemental Fig. 10). In particular, *AS1::LMI1* plants produced small bladeless leaves followed by arrested development (Supplemental Fig. 10C). These observations support the model predictions and highlight the importance of the precise regulation of *LMI1* for attaining correct leaf form.

To understand whether *LMI1* function is conserved between plants with different leaf shapes, we used an artificial microRNA (*amir-LMI1*) to silence expression of the orthologous *LMI1* gene in *Cardamine hirsuta*, a relative of *A. thaliana* with dissected leaves (Hay and Tsiantis 2006). Fully dissected leaves replaced stipules in these transgenic plants (Fig. 4C–F; Supplemental Fig. 11), indicating that *LMI1* function is conserved between crucifers with simple and dissected leaves. Furthermore, stipules of *amiR-LMI1;rco* plants were converted to simplified *rco-*like mutant leaves (Fig. 4G,H; Vlad et al. 2014). Therefore, *LMI1* and *RCO*, which are tandemly duplicated genes, largely function divergently in the leaf, consistent with their distinct expression domains (Vlad et al. 2014). Our findings also raise the possibility that evolutionary tinkering with the *LMI1* endoreduplication module, as described here, may underlie stipule transformations into leaf-like organs that are typical of many taxa (Darwin 1865; Sousa-Baena et al. 2018). For example, *Tendrilless* (*Tl*; pea *LMI1*) expression in pea leaves is absent from leafy stipules but present in bladeless tendrils, where it causes growth arrest, associated with increased endoreduplication (expression in tendrils) (Supplemental Fig. 12; also shown previously by Hofer et al. 2009). In conclusion, we demonstrated that spatially regulated expression of the LMI1 transcription factor influences organ proportions through an endoreduplication-mediated trade-off between cell and tissue growth. Our findings may help unify our understanding of the control of organ shape across multicellular eukaryotes. For example, in the developing vertebrate limb bud, the transcription factor GLI3R constrains digit size and number by limiting the pool of mesenchymal progenitors through directly repressing *Cdk6*, a regulator of the G1–S cell cycle transition (Lopez-Rios et al. 2012), highlighting the significance of local regulation of the cell cycle for controlling organ proportions. A future challenge will be to determine how broadly the growth trade-off that we identified here is used to control organ form and the degree to which it shaped organ diversity in different animals and plants (e.g., Sicard et al. 2014; Vlad et al. 2014; Andres et al. 2017).

## Materials and methods

Plants were cultivated in growth chambers under long day (16-h light/8-h dark) or short day (8-h light/16-h dark) conditions. *A. thaliana* and *C. hirsuta* were transformed using *Agrobacterium tumefaciens* floral dip transformations as in Hay et al. (2014). Confocal microscopy was performed with a SP8 upright laser scanning confocal microscope with a long working distance water immersion objective (AP 20×/0.8 M27; Zeiss). Statistical analyses were performed with Excel and the R package. Promoter sequence analyses and transcription factor-binding site search were performed with MEME/MAST package. A detailed description of the Materials and Methods is in the Supplemental Material.

## Supplementary Material

Supplemental Material
