# Supplementary material for: LMI1 homeodomain protein regulates organ proportions by spatial modulation of endoreduplication
Source: Genes Dev. 2018 Nov 1;32(21-22):1361–6. doi: 10.1101/gad.318212.118 (PMC6217736; doi:10.1101/gad.318212.118)
Supplement: Supplemental Material [file supp_32_21-22_1361__index.html]

Supplemental Material 

# LMI1 homeodomain protein regulates organ proportions by spatial modulation of endoreduplication

## Supplemental Material

- Supplemental\_Data.docx
