# Supplementary material for: LMI1 homeodomain protein regulates organ proportions by spatial modulation of endoreduplication
Source: Genes Dev. 2018 Nov 1;32(21-22):1361–6. doi: 10.1101/gad.318212.118 (PMC6217736; doi:10.1101/gad.318212.118)
Supplement: Supplemental Material [file supp_gad.318212.118_Supplemental_Data_.docx]

Title: **LMI1 homeodomain protein regulates organ proportions by spatial modulation of endoreduplication**

**Authors:** Francesco Vuolo^1^, Daniel Kierzkowski^1,‡^, Adam Runions^1^, Mohsen Hajheidari^1^, Remco A. Mentink^1^, Mainak Das Gupta^1^, Zhongjuan Zhang^1^, Daniela Vlad^2^, Yi Wang^1^, Ales Pecinka^3,†^, Xiangchao Gan^1^, Angela Hay^1^, Peter Huijser^1^ and Miltos Tsiantis^1^*

**Affiliations:**

^1^Deparment of Comparative Development and Genetics, Max Planck Institute for Plant Breeding Research, Carl-von-Linné-Weg 10, 50829 Cologne, Germany

^2^Department of Plant Sciences, University of Oxford, South Parks Road, Oxford OX1 3RB, UK.

^3^Department of Plant Breeding Genetics, Max Planck Institute for Plant Breeding Research, Carl-von-Linné-Weg 10, 50829 Cologne, Germany

^‡^Institute de Recherche en Biologie Végétale, Université de Montréal, 4101 Sherbrooke Est Montréal, QC H1X 2B2, Canada

^†^Institute of Experimental Botany of the Czech Academy of Sciences (IEB), Šlechtitelů 31, 77900 Olomouc-Holice, Czech Republic

^†,‡^ Current addresses

* Correspondence to: [tsiantis@mpipz.mpg.de](mailto:tsiantis@mpipz.mpg.de)

**Materials and Methods**

**Plant Material**

For phenotyping, crossing, ploidy analysis, DAPI staining, ChIP and SEM observations, plants were grown on soil in the greenhouse at 20°C, under long day conditions (16 hours of light at 20°C and 8 hours of darkness at 16°C). The different alleles used were *lmi1-2*, *bp-9*, *stm-2*, *cuc2-3*, *knat6-2*, *knat2-5*, *wox3-2* and *wee1-1*. Genetic crosses were performed in the greenhouse, in the order reported in the text. For Dexamethasone treatment, *LMI1::LMI1:GR* plants were grown on soil for 14 days at short day length conditions, then treated with Dexamethasone (DEX, dissolved in DMSO – 10 µM) and Cycloheximide (CHX – 10 µM) solution. They were sampled after 3h and an equivalent mock solution without DEX, but with CHX, was used as a control. Cotyledons and roots were removed during harvesting, before deep-freezing the plants in liquid nitrogen. For time-lapse analysis, plants were grown as described in (Barbier de Reuille et al. 2015).

**RNA extraction**

RNA was extracted from 14-day old seedlings using the Sigma Aldrich Plant Total RNA extraction kit. DNAse treatment was applied on-column during the extraction and subsequently tested with Actin primers to control for genomic DNA contamination. First strand cDNA synthesis was performed with the SuperScript® VILO™ cDNA Synthesis Kit (Invitrogen). qRT-PCR was performed using DNA-specific dye SYBR Green (Applied Biosystem) in a ViiA7 Real Time PCR machine (Applied Biosystems). Experiments were performed in three technical replicates, using three independent biological replicates. When a transgenic line was used, the three biological replicates consisted of three independent lines. Relative expression levels were determined as described in (Pfaffl 2001). Primers used in qPCR experiments are mentioned in Supplementary table 3.

**Confocal microscopy and time lapse experiments**

Confocal imaging was performed using an SP8 up-right laser scanning confocal microscope with a long working-distance water immersion objective (AP 20x/0.8 M27) (Zeiss). 4 excitation lasers were used, including an argon laser set at 514 nm to excite VENUS and at 488 nm to excite GFP. Images were collected with a 526-545 nm bandwidth filter for VENUS, 499-526 nm for GFP, 600-660 nm for PI and 660-749 nm for chlorophyll auto-fluorescence. The UV laser was set at 405 nm when performing DAPI staining. *LMI1::LMI1:VENUS* samples in Figure 1 were made by staining the shoots with 0.1% Propidium Iodide (Sigma), and holding the shoot in ½MS medium supplemented with 1.5% plant agar, 1% sucrose and 0.1% PPM (Plant Cell Technology). For time-lapse analyses the procedure was identical to the method reported in (Barbier de Reuille et al. 2015), except that the *lmi1-2* and wild-type plants for time-lapse experiments were expressing a plasma membrane marker (UBI::YFP:tag; (Yang et al. 2016)) to improve sample segmentation in MorphoGraphX. Excitation and detection settings for YFP were identical to those used for VENUS. For each genotype and analysis n ≥ 3.

**Cell lineage tracing**

We first segmented samples and subsequently calculated parent mappings where the correspondence of each cell to its daughters is determined between successive time-points as described in Barbier De Reuille et al. 2015. The parent mapping for each time-point is stored in a CSV file containing a label for each cell and the label of its parent in the previous time-point. These mappings were used directly to perform lineage tracing between successive time-points. Mappings spanning several time-points were created automatically using a python script and the mappings between successive days. For example, given parent mappings from 1-2 DAI and 2-3 DAI, the script finds the parent label at 1 DAI for each cell at 3 DAI. This is accomplished by using the label of a cell’s parent at 2DAI (stored in the 2-3 DAI parent map) to find its parent at 1 DAI using the 1-2 DAI parent map. Automation of the process through scripting reduces subjectivity and manual error in lineage analysis. To use the script copy the parent mappings between successive time-points (e.g. DAI1-2.csv, DAI2-3.csv and DAI3-4.csv) to the folder containing the script. The script can then be run from the command line as follows:

python lineage_script.py DAI1-2.csv DAI2-3.csv DAI3-4.csv DAI1-4.csv

Where lineage_script.py is the name of the script, and the file DAI1-4.csv specifies the CSV file where the parent mappings from 1-4 DAI will be stored. The script is available from the MorphoGraphX software page (www.MorphoGraphX.org).

The above information was also used to estimate founder cell initials as follows: For each sample, we identified the first time point where the clones comprising the stipule/lobe were largely restricted to the stipule and the initiation location could be visually identified. A lower bound on the number of initials was calculated by counting the number of cells at the first time-point whose contribution to the stipule could be confirmed at later time-points. The upper-bound was estimated by considering the number of additional cells that may have contributed to the stipule but whose contribution could not be confirmed due to incomplete segmentation of samples at all time points.

**Phenotypic analysis**

Leaf margin morphology and dissection indices were measured as described in (Vuolo et al. 2016). In Figure 3G and Supplemental Figure 1 the proximal and distal leaf domains, for which two separate dissection indices were calculated, were divided by a horizontal line traced below the most proximal serration. Leaf silhouettes were obtained by flattening the leaf on transparent adhesive film and scanning the leaf surface on black and white filters. For each genotype n = 10.

**Gene Ontology (GO) analysis and MEME/MAST motif analysis**

The list of Differentially Expressed Genes (DEGs) obtained from the RNA-seq experiment was produced as described in (Gan et al. 2016). For Gene Ontology analysis, we used the agriGO web tool, which generates a list of over-represented pathways in a hierarchical manner. A sub-group of the significantly enriched GO categories, related to cell-cycle and cell growth, were listed as in Figure 3B. The calculated p-value represents the significance of each category enrichment. The predicted LMI1 binding sites in the *WEE1* promoter (3 Kb) were detected by querying the *AtWEE1* upstream sequence (3 Kb) with a Position Weight Matrix (PWM) for the HD-ZIP I protein LMI1 (AtHB51) derived from Franco-Zorrilla et al and the Jaspar database (<http://jaspar.genereg.net/matrix/MA1375.1/>). Querying was done using the MEME/MAST discovery tool (Bailey and Gribskov 1998).

**Plant transformation**

Genetic transformation was performed using the floral dip technology with *Agrobacterium tumefaciens* strain GV3101 as reported (Clough and Bent 1998). T2 lines were obtained by self-pollination of T1s. Plants were selected on soil by spraying BASTA (Glufosinate-ammonium) 0.1%, or on Hygromycin plates (50 µM). In both cases, seeds were first stratified for 3 days at 4°C in the dark, and then moved to a climate chamber for germination.

**Chromatin Immuno-Precipitation**

Plants were grown on soil and harvested 14 days after germination. For each test or control line at least three biological replicates were used. 15-25 mg of tissue was collected and submerged in Crosslinking Buffer (1x PBS supplemented with 1% Formaldehyde), all while keeping the tissue on ice. Samples were then vacuum infiltrated for 5 minutes, followed by an additional 10 minutes. After releasing the pressure, Crosslinking Buffer was substituted with 1 M Glycine solution, and vacuum was applied for another 5 minutes. Glycine was removed, and the samples were washed with cold water. Samples were dried on absorbent paper, frozen in liquid nitrogen, and ground using mortar and pestle. For each 10 ml of pulverized tissue, 40 ml of Nuclei Isolation Buffer was added (50 mM Hepes; NaOH pH 7.4; 5 mM MgCl_2_; 25 mM NaCl; 2 mM EDTA; 2 mM DTT; 5% Sucrose; 30% Glycerol; 0.25% Triton X-100; PMSF 6 µl/ml; 400 µM PMFS; 10 µl/ml Sigma plant protease inhibitor). After mixing, the solution was filtered through a double nylon mesh (60 plus 20 μm), and centrifuged for 20 minutes at 3000 g at 4°C. The supernatant was discarded and the pellet resuspended in 30 ml Nuclear Wash Buffer (50 mM Hepes; NaOH pH 7.4; 20 mM MgCl_2_; 100 mM NaCl; 40% Sucrose; 40% Glycerol; 0.25% Triton X-100 and 2 mM DTT) and mixed at 4°C. Samples were centrifuged for 20 minutes at 3000 g at 4°C, with one repetition. Then the pellet was washed in Nuclear Wash Buffer without Triton X-100 and centrifuged for 20 minutes at 3000 g at 4°C. Pellets were resuspended in 500 μl TE (10 mM Tris-HCl, pH 8.0; 5 mM EDTA) containing 1% SDS and transferred to a 1.5 ml tube, incubated at 4°C, and gently mixed for 35 minutes. After dividing all the samples in single aliquots of 250 μl, chromatin sonication was performed with the Bioruptor sonicator for 13 cycles (30 sec ON / 30 sec OFF) at 4°C. Aliquots were transferred to a 2 ml tube and centrifuged 15 min at full speed at 4°C, the supernatant was then frozen in liquid nitrogen. GFP Trap® M beads were vortexed and dissolved in 10 mM Tris pH 7.5, 0.5 mM EDTA, 150 mM NaCl, added to the isolated chromatin and incubated under soft agitation at 4°C overnight. The magnetic beads for the test chromatin had anti-GFP fused to their surfaces. For the mock control half of the chromatin was incubated with magnetic beads fused to rat IgG. Beads were separated magnetically, in order to remove the supernatant and wash the beads with washing buffer 1 (0.1% SDS, 1% Triton-X, 2 mM EDTA, 20 mM Tris-HCl pH 7.5, 150 mM NaCl (filter sterilized)). This step was repeated twice with Washing Buffer 2 (0.1% SDS, 1% Triton-X, 2 mM EDTA, 20 mM Tris-HCl pH 7.5, 500 mM NaCl (filter-sterilized)) and Washing Buffer 3 (0.25 M LiCl, 1% IGEPAL-CA630, 1% deoxycholate, 1 mM EDTA, 10 mM Tris-HCl pH 7.5 (filter-sterilized)). A last washing step was performed with 10 mM Tris pH 7.5, 0.5 mM EDTA, after which captured proteins were eluted in 1.5 ml tubes with 0.5 ml Elution Buffer (50 mM Tris pH 8.0, 10 mM EDTA, 1% SDS) and incubated for 30 min at 65°C while shaking at a speed of 1300 rpm. Magnetic separation of the beads was performed in order to collect the supernatant. 15 μl of proteinase K (12 units, New England Biolabs p8107s) was added to each sample, and incubated for 5-6 hours at 37°C, followed by an O/N incubation at 65°C while mixing at 1300 rpm. 500 μl of Phenol/Chloroform/Isoamyl alcohol (25:24:1) was added to each sample, which was then vortexed for 30 seconds. Each sample was centrifuged for 5 min at 12000 g at room temperature, and the supernatant was transferred into a new tube. 500 μl of Chloroform/Isoamyl alcohol (24:1) was added and vortexed for 30 seconds, then samples were centrifuged for 5 min at 12000 g at room temperature. The top aqueous phase was transferred into a new tube. 50 μl of 3 M NaAc pH 5.2, 1250 μl of pure ethanol and 3 μl of Glycogen were added, then incubated for 2 hours at -20°C. Samples were centrifuged for 15 min at full speed at 4°C. Supernatant was removed and 1 ml of ice-cooled 70% ethanol was added to the pellet. Samples were centrifuged for 15 min at 14000 rpm at 4°C. The supernatant was discarded and the pellet air-dried at room temperature for at least 10 min. Finally, the pellet was dissolved in 40 μl DNase free water, to be sequenced or used as template in qRT experiments.

**Ploidy analysis**

Plants were grown on soil and harvested 14 days after germination. For each genotype, we selected equivalent leaf nodes of the same length, dissecting entire leaf primordia of approximately 5 mm. To isolate nuclei, fresh leaves were dissected and chopped into homogeneous suspension with a razor blade in 300 μl of Nuclei extraction buffer (part of CyStain UV Precise P kit, Sysmex). Subsequently, 700 μl of Staining buffer containing DAPI (part of CyStain UV Precise P kit, Sysmex) was added to the sample. Nuclei were separated from plant debris with CellTrics® filters (Sysmex) and the eluate was analyzed at the Partec Pas I system. Ploidy peaks were assigned manually and the Partec software quantified the percentage of nuclei contributing to each peak. Each ploidy class was quantified as a percentage of the combined C levels, calculated on the total amount of nuclei analyzed. n = 5 independent replicates were analyzed for each test case. For the *35S::LMI1* plants, 5 independent transgenic lines were used as 5 biological replicates.

**Scanning Electron Microscopy**

Plants were grown on soil, harvested and dissected to expose shoots or leaf nodes of interest. Fixation was performed under vacuum in FAEG (4% Formaldehyde; 4% Acetic acid; 80% of Absolute Ethanol; 0,01% Glutaraldehyde) for 10 minutes. After releasing the vacuum, new fixative was replaced once and samples were kept O/N at 4°C. Samples were then dehydrated by replacing the fixative first with ethanol 60%, then with ethanol 85% and finally with absolute ethanol. Critical point drying of the sample was performed in absolute ethanol, with 23 slow cycles at 35°C and a pressure of 1200 psi in the LEICA EM CPD300 machine. Samples were then positioned on metal platforms and sputter-coated with platinum in the Leica 7620 system. Observation and photographs were performed with a Scanning Electron Microscope 40VP Supra from Zeiss. To confirm any observed phenotypes, at least 10 biological replicates were analyzed.

**DAPI staining of polytene structures**

The polytene regions are cable-like agglomerates of sister chromatids resulting from endocycles (Nagl 1981). It is possible to visualize them in the cell nucleus via DAPI staining (Breuer et al. 2007). To analyze polytene regions of nuclei we fixed mature leaves dissected from *lmi1-2* and wild-type plants. Fixation was performed in 3.7% Formaldehyde in PBST (PBS solution with 0.1% Tween) under vacuum for 10 minutes. After releasing the vacuum, samples were washed in fresh PBST and stored O/N at 4°C. Samples were washed with water, immersed in Clear-see solution (Kurihara et al. 2015) and kept for one day at room temperature. Clear-see solution was removed and samples were fixated under vacuum in DAPI staining solution (0.25 mg/mL, 5% DMSO in PBST) for 15 minutes. Samples were then stored in the dark at 4°C O/N. The next day the samples were observed at the confocal microscope, using a UV laser set at 405 nm to excite DAPI, n ≥10. For each leaf we focused on distal margin cells and stipules of Col-0/*lmi1-2*. We performed a volumetric analysis of the nuclei from these cells, to count polytene regions. The Z-stacks were processed with MorphographX to produce the photographs in Supplemental Figure 6.

**Allometry analysis**

In case of a simple allometric comparison between two organs, x and y, the growth of y is expected to be proportional to x^α^, where α is the *differential growth constant* (or *growth ratio*) between y and x. To compare the growth rate of the leaf to that of the stipule/ectopic leaf, the growth of the leaf and stipule/ectopic leaf were measured (on the basis of SEM samples, n ≥ 5 for each stage) and assigned the values x and y, respectively. The values were plotted on a double logarithmic grid and fitted to a power equation (x ∞ y^α^). The resulting slope was used to calculate the differential growth ratio.

**Constructs and cloning strategies**

Plasmids containing synthetized *AthWEE1* cds, *AthLMI1:VENUS*, *AthCC52A* and *amiR-ChLMI1i* sequences were ordered from GENEWIZ. *WEE1*, *CCS52A* and *LMI1:VENUS* coding sequences were synthetized with 5’-XmaI and 3’-KpnI (for the first two cds) and 5’-Xma and 3’-BamHI (for *LMI1:VENUS*) restriction sites at their flanks, in order to clone them into the pBJ36 vector, downstream of the *LMI1* promoter, previously inserted with 5’-PstI and 3’-XmaI restriction sites. For *LMI1::LMI1:GR* lines, the *LMI1:GR* cds was synthesized by GenScript, fusing a glucocorticoid receptor domain to the *LMI1* ORF, and subcloning it under the *LMI1* promoter using Xma-BamHI restriction. In all cases, the whole transgenic cassette was then subcloned into a pMLBART vector using NotI-mediated restriction. The *amiR-LMI1i* sequence was synthesized with 5’-KpnI and 3’-XbaI restriction sites and inserted into the pART7 vector. Then the whole cassette was subcloned into pMLBART with NotI-mediated restriction. pMLBART was used as a final vector for plant transformation.

**Genotyping strategies**

Genotyping strategies for the alleles used in this study were the ones described for *lmi1-2* (Vlad et al. 2014), for *cuc2-3* (Hibara et al. 2003), for *wox3-2* (Zhang et al. 2017), for *stm-2* (Clark et al. 1996), and for *knat2-5*, *knat6-1* and *bp-9* (Ragni et al. 2008). Genotyping primers are mentioned in Supplemental table 3.

**Construction of the model for endoreduplication and organ size**

To examine the relation between cellular behaviours and organ size we constructed a minimial cell-population model simulating proliferation, endoreduplication and differentiation (Figure 4a-b, Supplemental Figure 9). Our model is a variant of those proposed previously (Roeder et al. 2010; Kawade and Tsukaya 2017). In the model, proliferative cells were represented by a single population (assumed to be *2C*), whereas endoreduplicating cells were divided into sub-populations based on their ploidy ($2C,4C,8C,\cdots$ ; Supplemental Figure 9b). Transitions between populations accounted for by the model are shown in Supplemental Figure 9a-b. In simulations, the timing of proliferative activity and differentiation were fixed while the window of active endoreduplication was varied (Figure 4a), allowing the effect of endoreduplication on organ size to be evaluated. Simulations are available upon request and were implemented and visualized in lpfg (Karwowski and Prusinkiewicz 2003). Parameter values for all simulations are reported in Supplemental table 2.

Proceeding more formally, $P$ denotes the number of proliferative cells, and $E_{i}$ denotes the number of endoreduplicative cells which have completed $i$ endocycles (i.e. the number of $2^{i}C$ endoreduplicating cells). Temporal dynamics are captured by the following ordinary differential equations, with the rate constants introduced in Supplemental Figure 9:

$\frac{dP}{dt}=R_{s}P-R_{e}P,$ (1)

$\frac{dE_{0}}{dt}=R_{e}P-R_{s}E_{0},$ (2)

$\frac{dE_{i}}{dt}=R_{s}E_{i-1}-R_{s}E_{i}.$ (3)

where *i*>0 in Eq. 3. These equations account for the transitions between populations shown in Supplemental Figure 9a-b. To provide initial conditions for our simulations we assume that $P(0)=P_{0}$ and $E_{i}(0)=0$ (i.e. initially all cells are proliferative).

Active proliferation and endoreduplication are limited to a given time window (Figure 4a). As a simplification, this activity is captured by step functions. Differentiation is incorporated by assuming that proliferation and endoreduplication ceases at time $T_{diff}$. Proliferative activity is limited to early stages of organ development by setting $R_{s}=0$ in Eq. 1 when $t>T_{prolif}$ or $t>T_{diff}$. Active endoreduplication is restricted to a given window $[T_{endo0},T_{endo1}]$ by setting $R_{e}=0$ and $R_{s}=0$ when or $t>T_{diff}$. To focus on effects stemming from the relative timing of proliferation and endoreduplication, as opposed to their relative duration, the length of the activation window is the same for both processes (i.e. $T_{prolif}=T_{endo1}-T_{endo0}$). Altogether, this formulation provides a trade-off between simplicity and expressiveness of models, allowing us to qualitatively examine interactions between proliferation and endoreduplication while limiting the number of free model parameters.

To relate ploidy levels to relative increases in cell size we assume that each round of endoreduplication increases final cell area by a factor of 1.5 (as reported for leaf epidermal cells in (Kawade and Tsukaya 2017)). From this we can derive the predicted size increase *A* of the entire population based on the sub-populations simulated by the model:

$A=\frac{P+\sum_{i=0}^{n} 1.5^{i}E_{i}}{P_{0}}.$ (4)

Here, the first term in the numerator accounts for proliferative cells $P$, which differentiate before entering an endoreduplicative state, and the second term accounts for endoreduplicating cell populations $E_{i}$. To access the relative increase in size we divide by $P_{0}$.

**RNA *in situ* hybridization**

*Pisum sativum* wild-type plants were grown for two weeks at 23 °C in short day conditions. Plant shoots were fixated following the procedure described in (Rast-Somssich et al. 2015). Sections were 8 microns and *in-situ* probes were generated from a synthetic template. The digoxigenin-labelled antisense RNA probes to *P. sativum TENDRIL-LESS (TL)* were generated from two both 485 nt long synthetic DNA templates (GenScript, Hong Kong), covering the entire TL-cds plus 116 and 140 nts of resp. up- and downstream exon sequences (Genbank entry EU938525.1). For in vitro transcription, a 25 nt T7 RNA polymerase binding motif was added to their 3' ends.

**Statistics and reproducibility.**

Statistical analyses were performed in the R environment for all box-plots and in Microsoft excel for all other graphs. Bar graphs were analyzed with a two-tailed Student’s t-test in Microsoft Excel; we reported three different layers of statistical significance below the following thresholds: 0.05; 0.01 and 0.001. When variance was strongly asymmetric round the mean the K-S test was used. All error bars in the bar graphs represent mean ± sem. Sample size is reported in each figure legend. In box-plots, the center is the median, whilst the lower and upper box lines represent the 25th and 75th percentile, respectively. Dots represent individual samples. Individual biological samples are stated as n. All qRT-PCR reactions were performed using at least three biological replicates and were independently replicated three times. All experiments that involved the use of transgenic lines were performed on at least 5 independent T1 or T2 lines. Paired-end reads were aligned to the reference genome TAIR10 for *A. thaliana* using TopHat2 with parameters “--max-multihits 10 --coverage-search --microexon-search --mate-std-dev 40 --library-type fr-secondstrand --max-intron-length 30000”. Raw read counts per gene were quantified with HTSeq v0.5.4p1 (http://www-huber.embl.de/users/anders/HTSeq/) using the " -t CDS -s reverse" option. Differential expression between samples from the same species was determined using DESeq. We found the most sensitive parameter settings for the function *estimateDispersions* were method="blind", and sharingMode="fit-only.

**Data availability**

All lines and data generated in this study are available from the corresponding author upon request. High throughput RNA-seq data generated during this study are publicly available: <http://chi.mpipz.mpg.de/misc/lmides.html>

**References**

Bailey TL, Gribskov M. 1998. Combining evidence using p-values: application to sequence homology searches. *Bioinformatics* **14**: 48-54.

Barbier de Reuille P, Routier-Kierzkowska AL, Kierzkowski D, Bassel GW, Schupbach T, Tauriello G, Bajpai N, Strauss S, Weber A, Kiss A et al. 2015. MorphoGraphX: A platform for quantifying morphogenesis in 4D. *Elife* **4**: 05864.

Breuer C, Stacey NJ, West CE, Zhao Y, Chory J, Tsukaya H, Azumi Y, Maxwell A, Roberts K, Sugimoto-Shirasu K. 2007. BIN4, a novel component of the plant DNA topoisomerase VI complex, is required for endoreduplication in Arabidopsis. *Plant Cell* **19**: 3655-3668.

Clark SE, Jacobsen SE, Levin JZ, Meyerowitz EM. 1996. The CLAVATA and SHOOT MERISTEMLESS loci competitively regulate meristem activity in Arabidopsis. *Development* **122**: 1567-1575.

Clough SJ, Bent AF. 1998. Floral dip: a simplified method for Agrobacterium-mediated transformation of Arabidopsis thaliana. *Plant J* **16**: 735-743.

Gan X, Hay A, Kwantes M, Haberer G, Hallab A, Ioio RD, Hofhuis H, Pieper B, Cartolano M, Neumann U et al. 2016. The Cardamine hirsuta genome offers insight into the evolution of morphological diversity. *Nat Plants* **2**: 16167.

Hibara K, Takada S, Tasaka M. 2003. CUC1 gene activates the expression of SAM-related genes to induce adventitious shoot formation. *Plant J* **36**: 687-696.

Karwowski R, Prusinkiewicz P. 2003. Design and implementation of the L+ C modeling language. *Electronic notes in theoretical computer science* **86**: 134-152.

Kawade K, Tsukaya H. 2017. Probing the stochastic property of endoreduplication in cell size determination of Arabidopsis thaliana leaf epidermal tissue. *PLoS One* **12**: e0185050.

Kurihara D, Mizuta Y, Sato Y, Higashiyama T. 2015. ClearSee: a rapid optical clearing reagent for whole-plant fluorescence imaging. *Development* **142**: 4168-4179.

Nagl W. 1981. Polytene Chromosomes of Plants. *Int Rev Cytol* **73**: 21-53.

Pfaffl MW. 2001. A new mathematical model for relative quantification in real-time RT-PCR. *Nucleic Acids Res* **29**: e45.

Ragni L, Belles-Boix E, Gunl M, Pautot V. 2008. Interaction of KNAT6 and KNAT2 with BREVIPEDICELLUS and PENNYWISE in Arabidopsis inflorescences. *Plant Cell* **20**: 888-900.

Rast-Somssich MI, Broholm S, Jenkins H, Canales C, Vlad D, Kwantes M, Bilsborough G, Dello Ioio R, Ewing RM, Laufs P et al. 2015. Alternate wiring of a KNOXI genetic network underlies differences in leaf development of A. thaliana and C. hirsuta. *Genes Dev* **29**: 2391-2404.

Roeder AH, Chickarmane V, Cunha A, Obara B, Manjunath BS, Meyerowitz EM. 2010. Variability in the control of cell division underlies sepal epidermal patterning in Arabidopsis thaliana. *PLoS Biol* **8**: e1000367.

Saddic LA, Huvermann B, Bezhani S, Su Y, Winter CM, Kwon CS, Collum RP, Wagner D. 2006. The LEAFY target LMI1 is a meristem identity regulator and acts together with LEAFY to regulate expression of CAULIFLOWER. *Development* **133**: 1673-1682.

Vlad D, Kierzkowski D, Rast MI, Vuolo F, Dello Ioio R, Galinha C, Gan X, Hajheidari M, Hay A, Smith RS et al. 2014. Leaf shape evolution through duplication, regulatory diversification, and loss of a homeobox gene. *Science* **343**: 780-783.

Vuolo F, Mentink RA, Hajheidari M, Bailey CD, Filatov DA, Tsiantis M. 2016. Coupled enhancer and coding sequence evolution of a homeobox gene shaped leaf diversity. *Genes Dev* **30**: 2370-2375.

Yang W, Schuster C, Beahan CT, Charoensawan V, Peaucelle A, Bacic A, Doblin MS, Wightman R, Meyerowitz EM. 2016. Regulation of Meristem Morphogenesis by Cell Wall Synthases in Arabidopsis. *Curr Biol* **26**: 1404-1415.

Zhang Z, Tucker E, Hermann M, Laux T. 2017. A Molecular Framework for the Embryonic Initiation of Shoot Meristem Stem Cells. *Dev Cell* **40**: 264-277 e264.


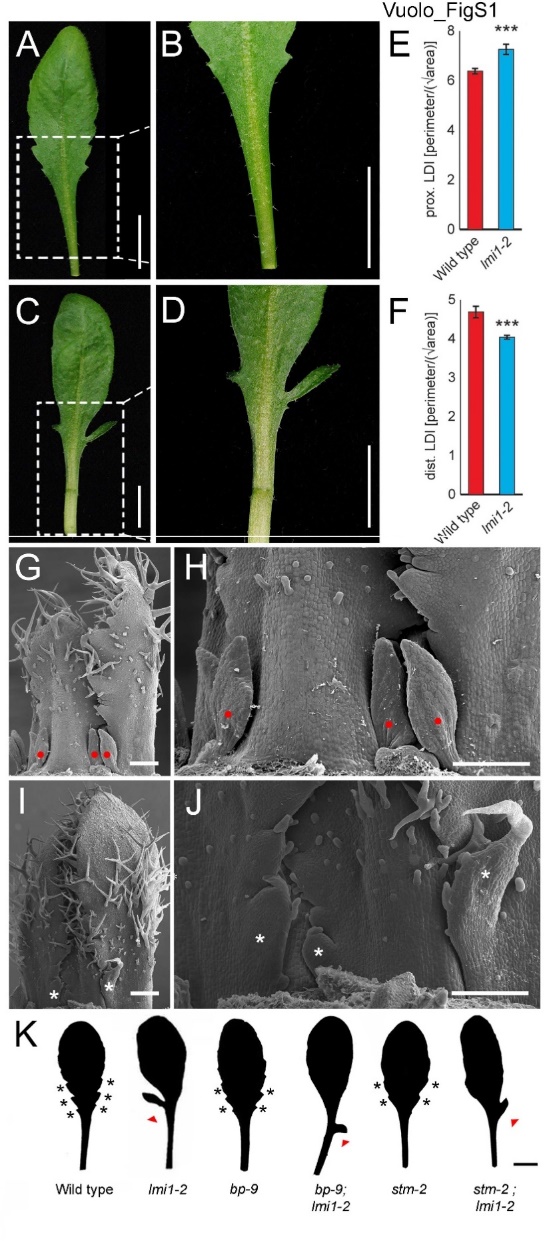


**Supplemental Figure 1. Lobes replace stipules in *lmi1-2* leaves. (A-D)** Wild-type (A-B) and *lmi1-2* (C-D) leaf 11. b and d are blow-ups of a and c, respectively, n = 25. Scale bar: 1 cm. **(E-F)** Leaf Dissection Index (LDI) of the proximal (E) and distal (F) part of wild-type (red) and *lmi1-2* (blue) leaf 11, n = 10, mean ± sem, Student’s t-test *: P<0.001. The proximal and distal domains were defined as the leaf area below and above the sinuses at the boundary with the petiole. **(G-J)** Scanning electron micrographs of wild-type (G-H) and *lmi1-2* (I-J) shoot apices during late vegetative phase. H and J are blown-ups of G and I, respectively. Red dots: stipules, asterisks: ectopic lobes. Note the trichomes on ectopic lobes, indicating leaf identity, n≥30. Scale bar: 100 µm. **(K)** Silhouettes of leaf 11 in wild type and mutants. Scale bar: 1 cm (A-D, K), 100 µm (G-J). n = 25.


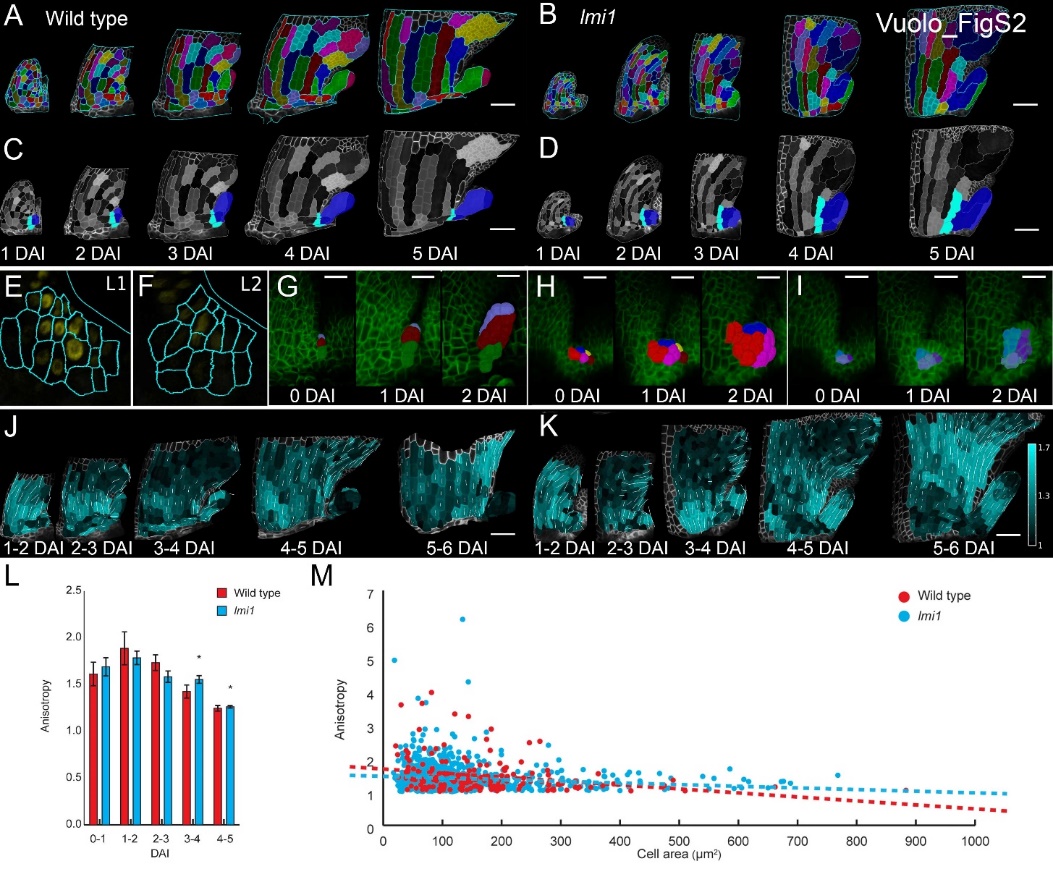


**Supplemental Figure 2. *LMI1* expression in L1 and L2 cell layers of the stipule represses tissue growth and anisotropy in the stipule. (A-D)** Clonal analysis (A-B) and cell lineage tracing of the stipule (dark blue) and stipule boundary (cyan) (C-D) in wild-type (A,C) and *lmi1-2* (B,D) leaf 11. More and larger clonal sectors are present in *lmi1-2* than in wild type, suggesting that *LMI1* restricts the number of stipule initials, and the growth of stipule tissue. Scale bars: 50 µm. **(E-F)** Confocal laser scanning micrographs of *LMI1::LMI1:VENUS* expression in L1 and L2 cell layers of stipule initials. **(G-I)** 3-D multilayer clonal analysis of wild-type (G) and *lmi1-2* (H) L1 layer cells, and *lmi1-2* L2 layer cells (I) at 0, 1 and 2 DAI. Scale bars: 20 µm, n = 5. **(J-K)** Tissue anisotropy quantified from leaf 11 time-lapse series in wild type (J) and *lmi1-2* (K). Anisotropy measured in a scale from black to blue. In panels A-K, cells were outlined by PM-YFP expression and 3 different samples were imaged under time lapse conditions at successive DAI for each analysis, n ≥ 3. **(L)** Box plot showing cell anisotropy is higher in *lmi1-2* lobes (blue) than wild-type stipules (red), n >50 cells in 3 biological replicates, mean ± SD, Student’s t-test *: P<0.05. **(M)** Correlation between cell area and anisotropy values pooled for all DAI in wild-type stipule cells (red) and *lmi1-2* lobe cells (blue). Linear regressions are shown by dashed lines.


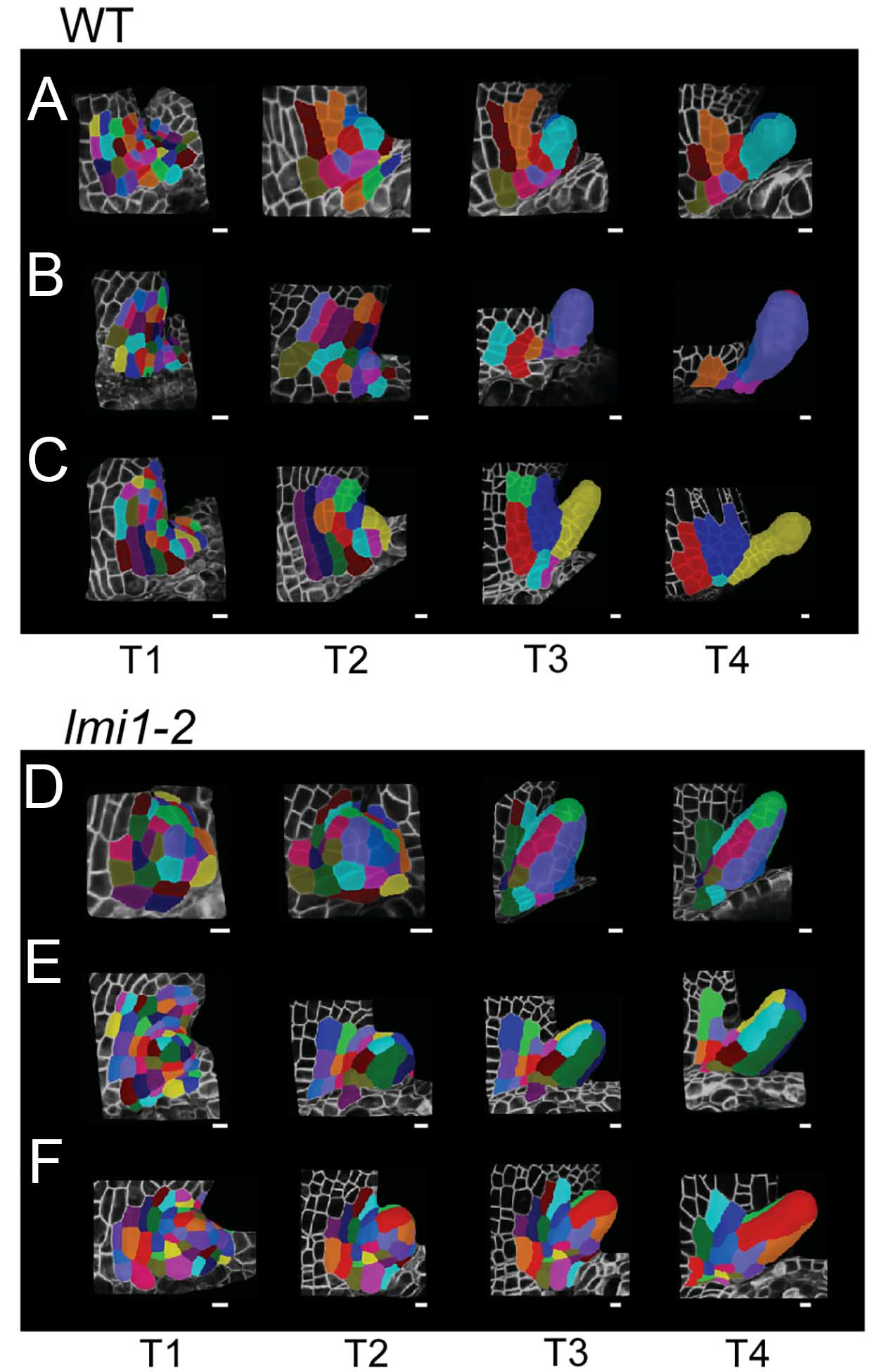


**Supplemental Figure 3. *LMI1* restricts the number of stipule initial cells.** Cell lineage tracing of **(A-C)** wild-type stipules and **(D-F)** *lmi1-2* lobes at four consecutive developmental stages (T1 to T4), spanning intervals of approximately 72h (A,E), 96h (B,F) and 120h (C,D). Single cell clones are marked with distinct colors. Wild-type stipules descend from fewer initials than *lmi1-2* lobes (compare how many colors compose the final stipules in both genotypes)*.* Three replicates are shown for each genotype, in addition to the samples shown in Figure 2 and Supplemental Figure 2. Scale bar: 10 µm.


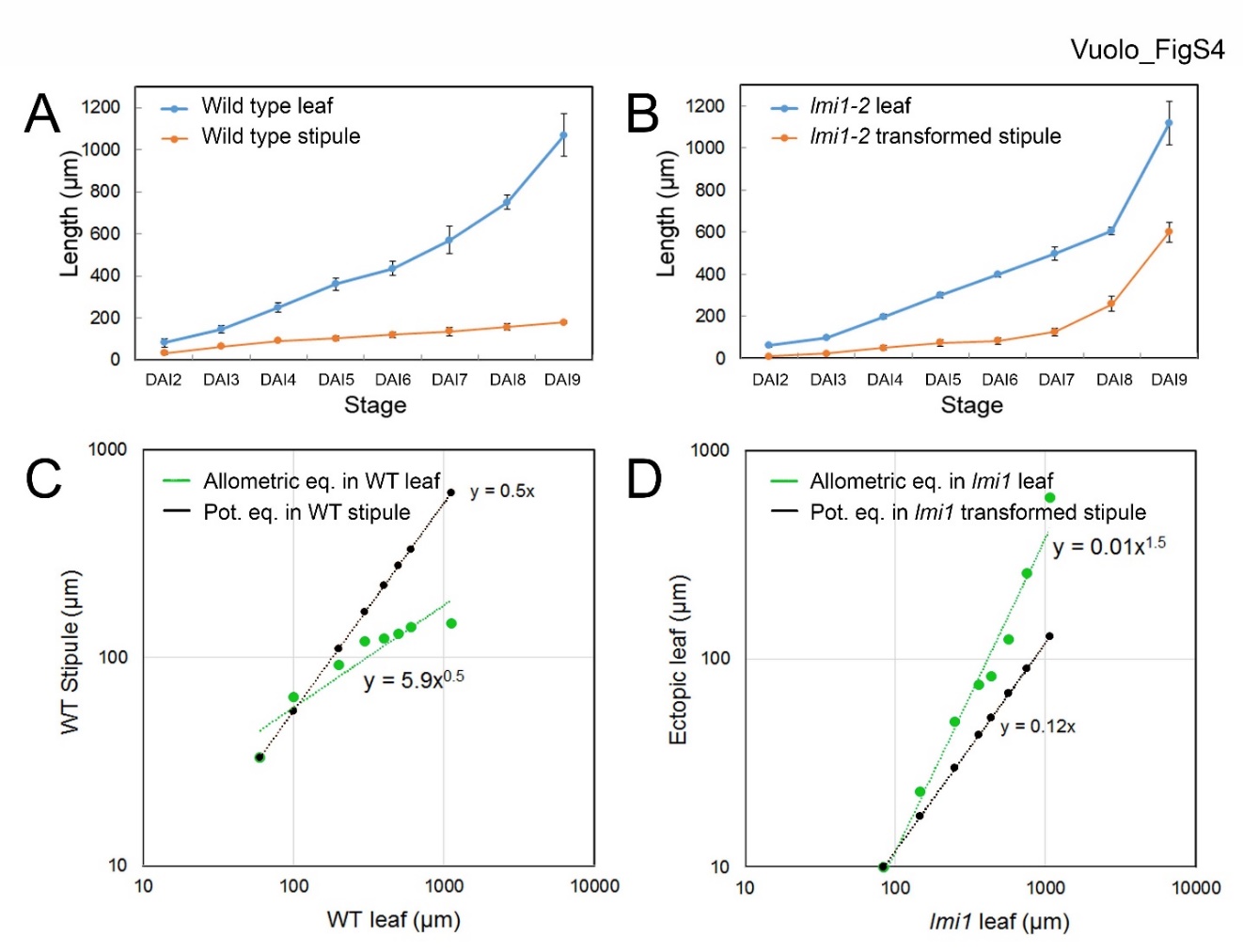


**Supplemental Figure 4. *LMI1* regulates the allometric proportions of stipules and leaf. (A-B)** Lengths of the wild type leaf and stipule (A) compared to the leaf and ectopic lobe in *lmi1-2* (B) on successive days after leaf initiation (DAI), n ≥ 5, mean ± SD. **(C-D)** Analysis of differential growth rates between the wild-type leaf and stipule (C) and between the *lmi1-2* leaf and ectopic lobe (D). The values of lengths were fitted to the allometric equation y=x^α^, where α represents the differential growth ratio between two growing regions, x (wild-type leaf, C; and *lmi1-2* leaf, D) and y (wild-type stipule, C; and *lmi1-2* ectopic lobe, D). Black circles represent the hypothetical values (potential equation, Pot. eq.) for x and y if the WT leaf and stipule (C) or the *lmi-1* leaf and ectopic lobe (D) had grown at the same rates (starting from the initial size). The green circles represent the actual values during growth progression. The slope of the green plot represents the growth ratio between the leaf and the stipule/ectopic leaf. The length measurements were taken from scanning electron micrographs.


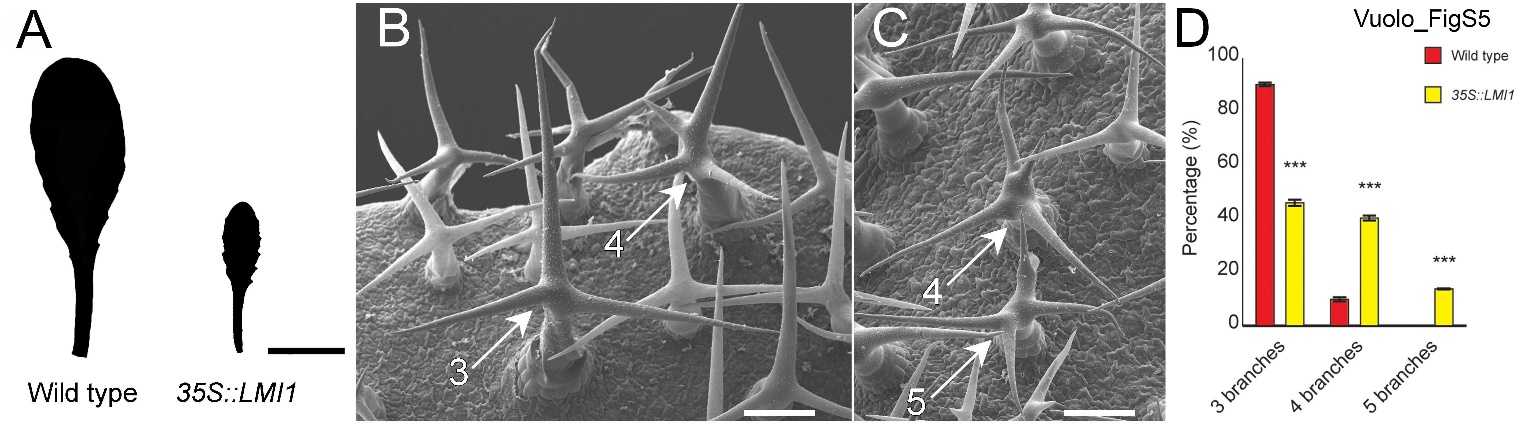


**Supplemental Figure 5. *35S::LMI1* leaves show reduced size and increased trichome branching. (A)** Silhouette of leaf 11 in wild type and *35S::LMI1*. Scale bar: 1 cm. **(B-C)** Scanning electron micrographs of leaf trichomes in wild type (B) and *35S::LMI1* (C). Numbers with arrows show the number of branches for the indicated trichomes. Scale bar: 100 µm. **(D)** Plot showing the percentage of 3-, 4- and 5-branched trichomes on wild-type (red) and *35S::LMI1* (yellow) leaves, n > 10, mean ± SD, Student’s t-test ***: P < 0.001. Leaf and trichome images represent consistent phenotypes observed in 10 independent transgenic lines.


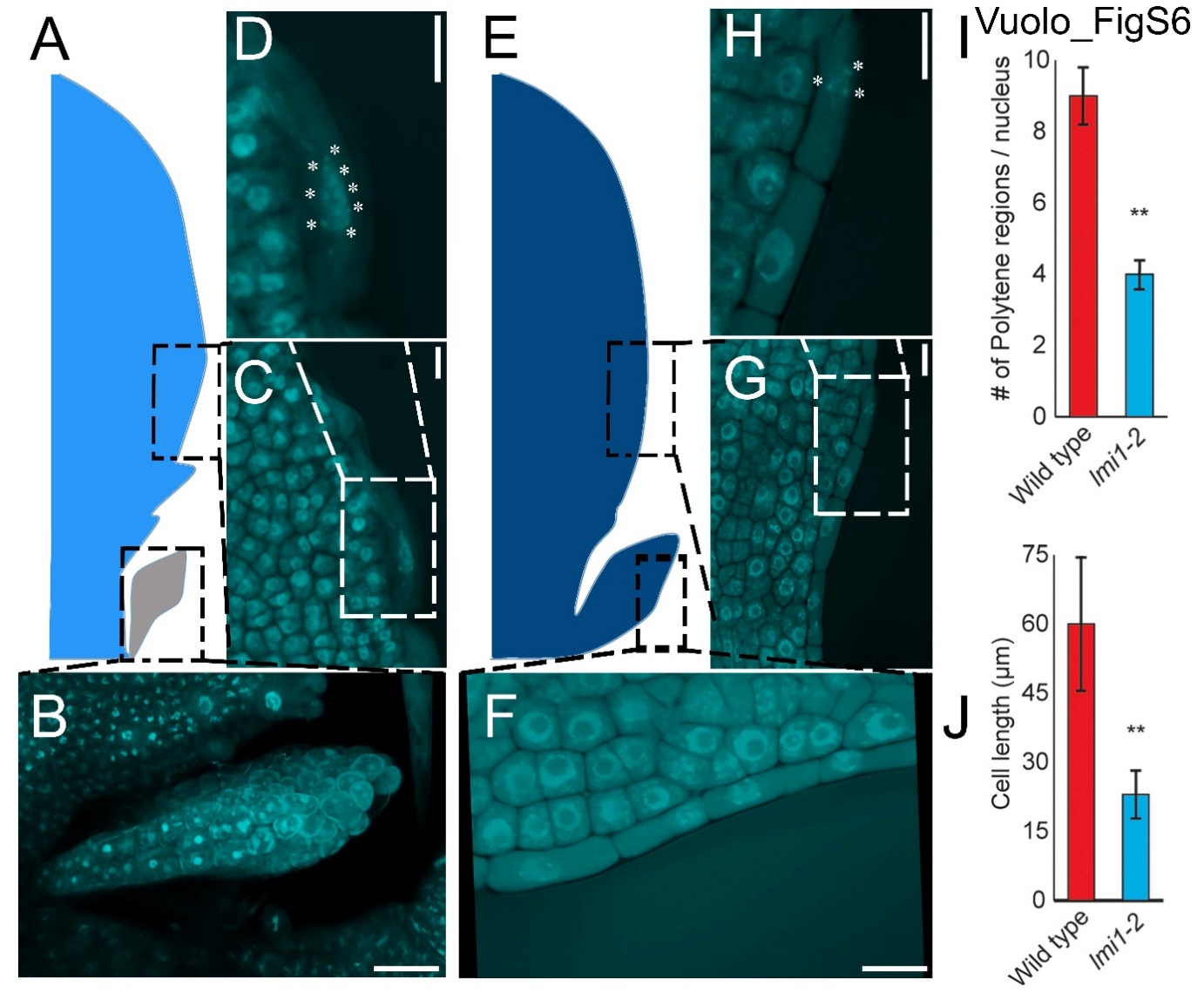


**Supplemental Figure 6. Activation of endoreduplication by LMI1 regulates margin and stipule cell size. (A,E)** Cartoons of wild-type (A) and *lmi1* (E) leaves indicating the location of confocal laser scanning micrographs of DAPI-stained cells in the stipule **(B)** and margin **(C-D)** of wild type, and in the ectopic lobe **(F)** and margin **(G-H)** of *lmi1*. (D) and (H) are blow-ups of the dashed areas marked in (C) and (G), respectively. White asterisks represent polytene regions. Scale bars: 10 µm (B,F), 5 µm (C-D, G-H). **(I)** Polytene regions and **(J)** cell length analyses in margin cells of wild-type (red) and *lmi1-2* (blue) leaves, n ≥ 10, mean ± SD, Student’s t-test **: P < 0.01.


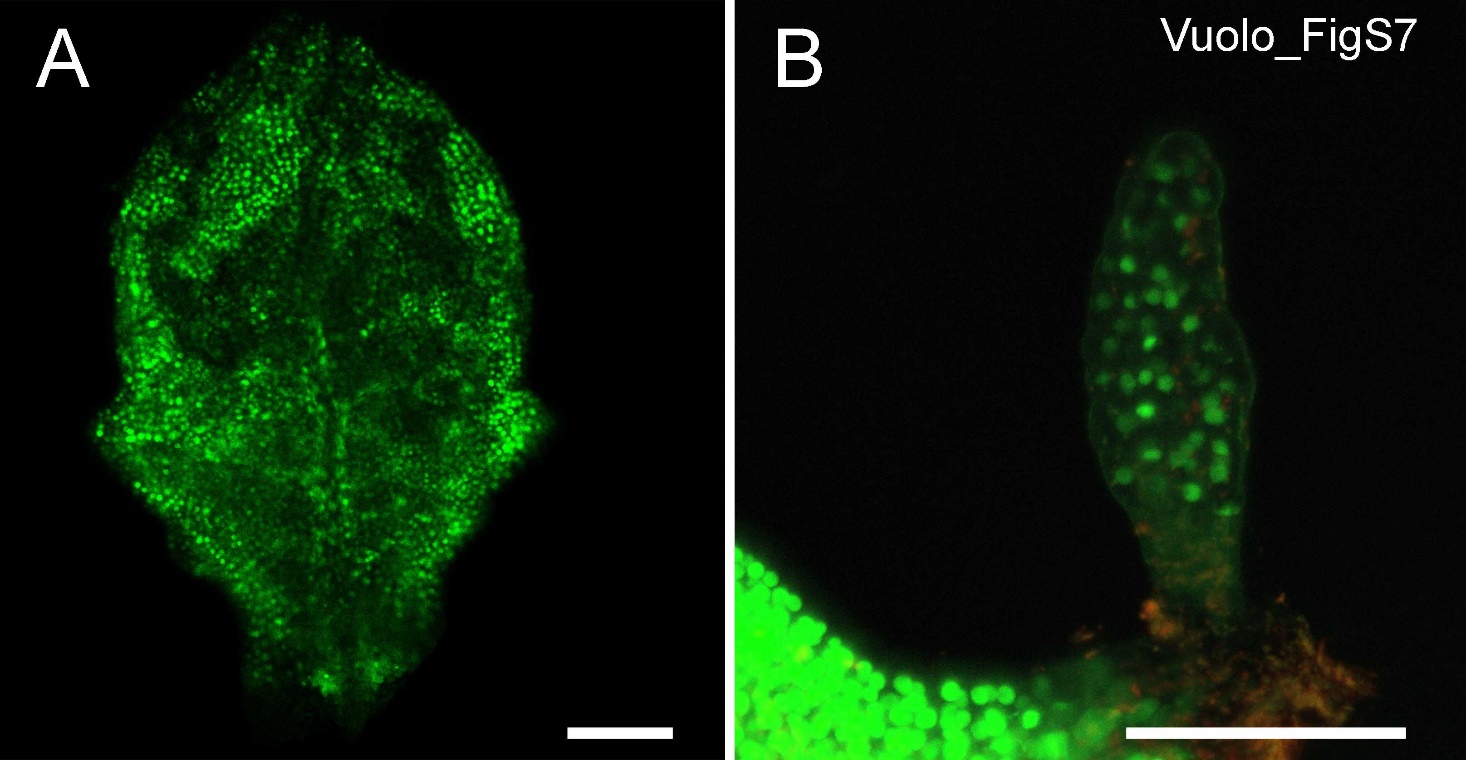


**Supplemental Figure 7. *WEE1* expresses broadly in shoots, leaves and stipules in *A. thaliana*. (A-B)** Confocal laser scanning micrographs of *AtWEE1::3xGFP* expression in leaves (A) and stipules (B). Images are shown as maximal projections and represent a consistent expression pattern found in 15 independent transgenic lines. Scale bar: 50 µm.


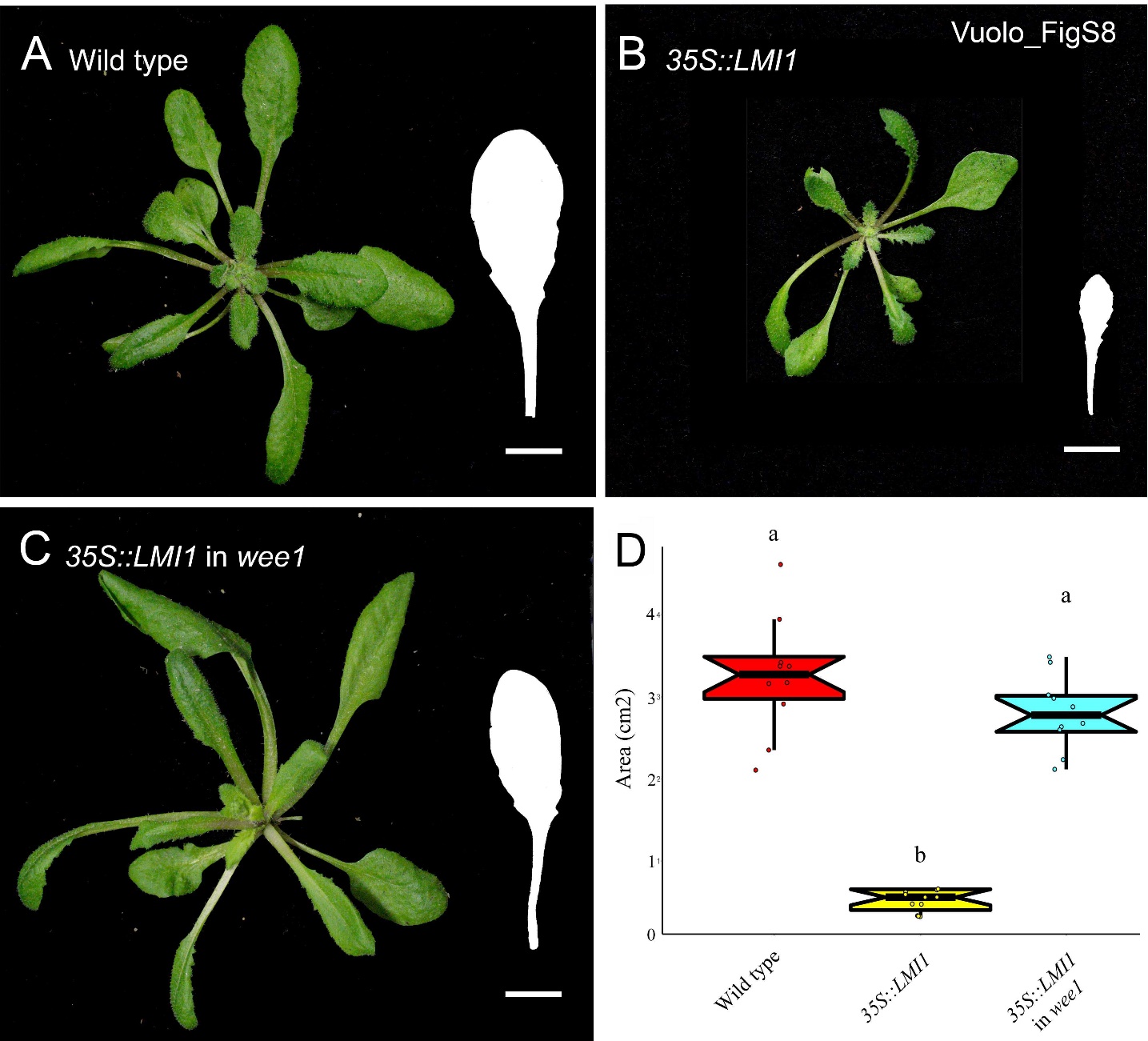


**Supplemental Figure 8. *WEE1* is necessary to mediate *LMI1*-induced growth repression. (A-C)** Digital photographs of (A) wild type, (B) *35S::LMI1* in wild-type background and (C) *35S::LMI1* in *wee1-1* background. In each panel, a representative leaf 6 silhouette (in white) is shown. Scale bar 1 cm. **(D)** Box plot graph showing the leaf area distribution in the above-mentioned transgenic lines. Letters mark significant differences between groups as indicated by ANOVA and post-hoc Tukey’s test (p<0.01), n = 10 independent transgenic lines.


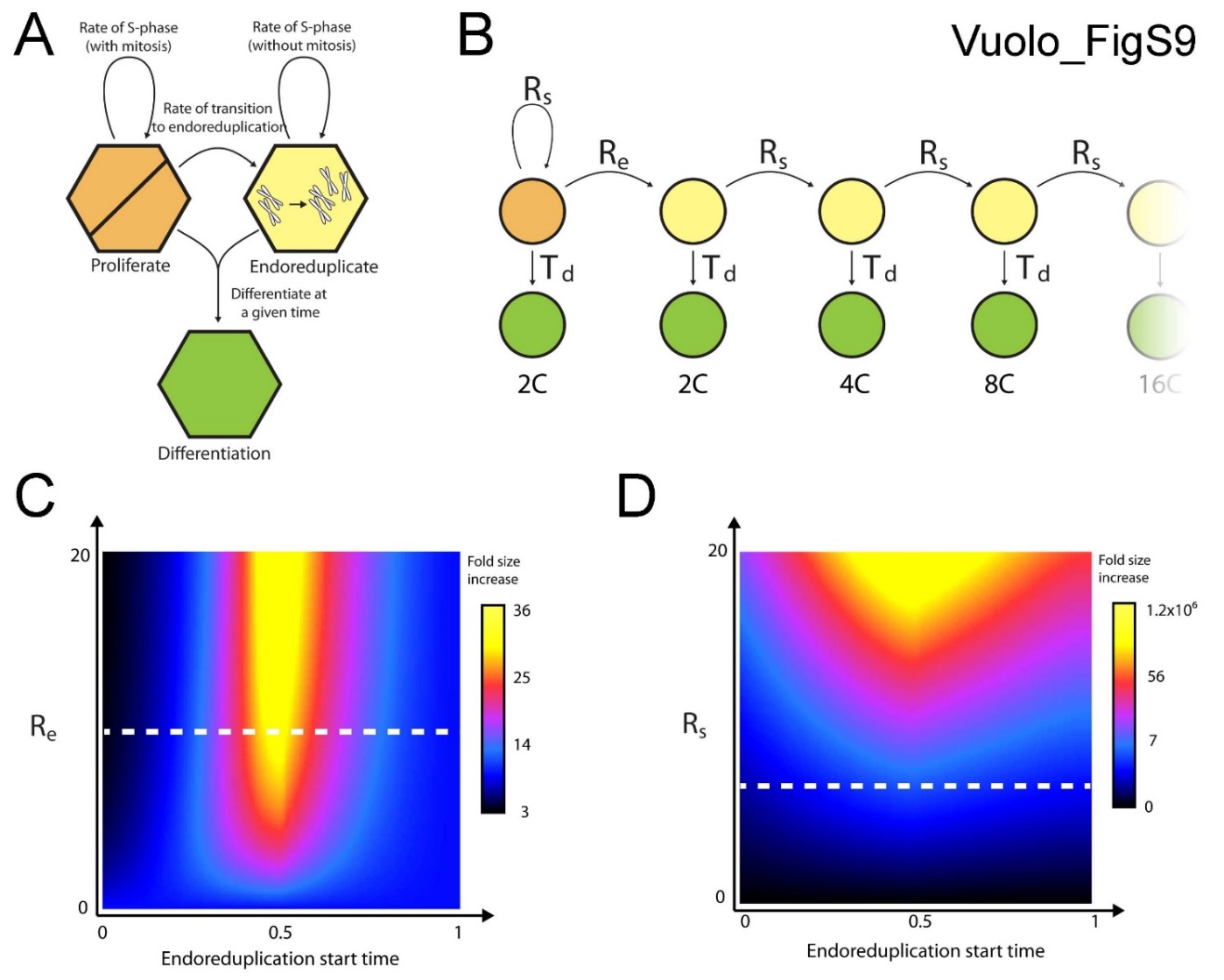


**Supplemental Figure 9. Cell population model, accounting for the timing and rates of endoreduplication and proliferation, recapitulates organ size variation. (A)** Proliferative and endocycling cells undergo s-phase at rate *R_s_*, proliferative cells switch to endocycling at a rate *R_e_*, and all cells differentiate at a given time *T_d_*. Proliferation increases the population of proliferative cells; endocycling increases ploidy. **(B)** Cell populations (circles) and transitions between populations (arrows) in the model presented in (A). Proliferative cells (orange) are 2C and proliferate at rate *R_s_* during the window of proliferation. During the window of endoreduplication, proliferative cells transition to endoreduplication at rate *R_e_.* Endoreduplicating cells (yellow) endocycle at rate *R_s_*, causing their ploidy to double. At time *T_d_* all cells differentiate (green); proliferation and endoreduplication stop. **(C-D)** Heat maps showing organ size variation as endoreduplication start-time (x-axis) is varied together with *R_e_* (y-axis, C), or *R_s_* (y-axis, D). The dashed line indicates the cross-sections corresponding to the plot shown in Figure 4B.


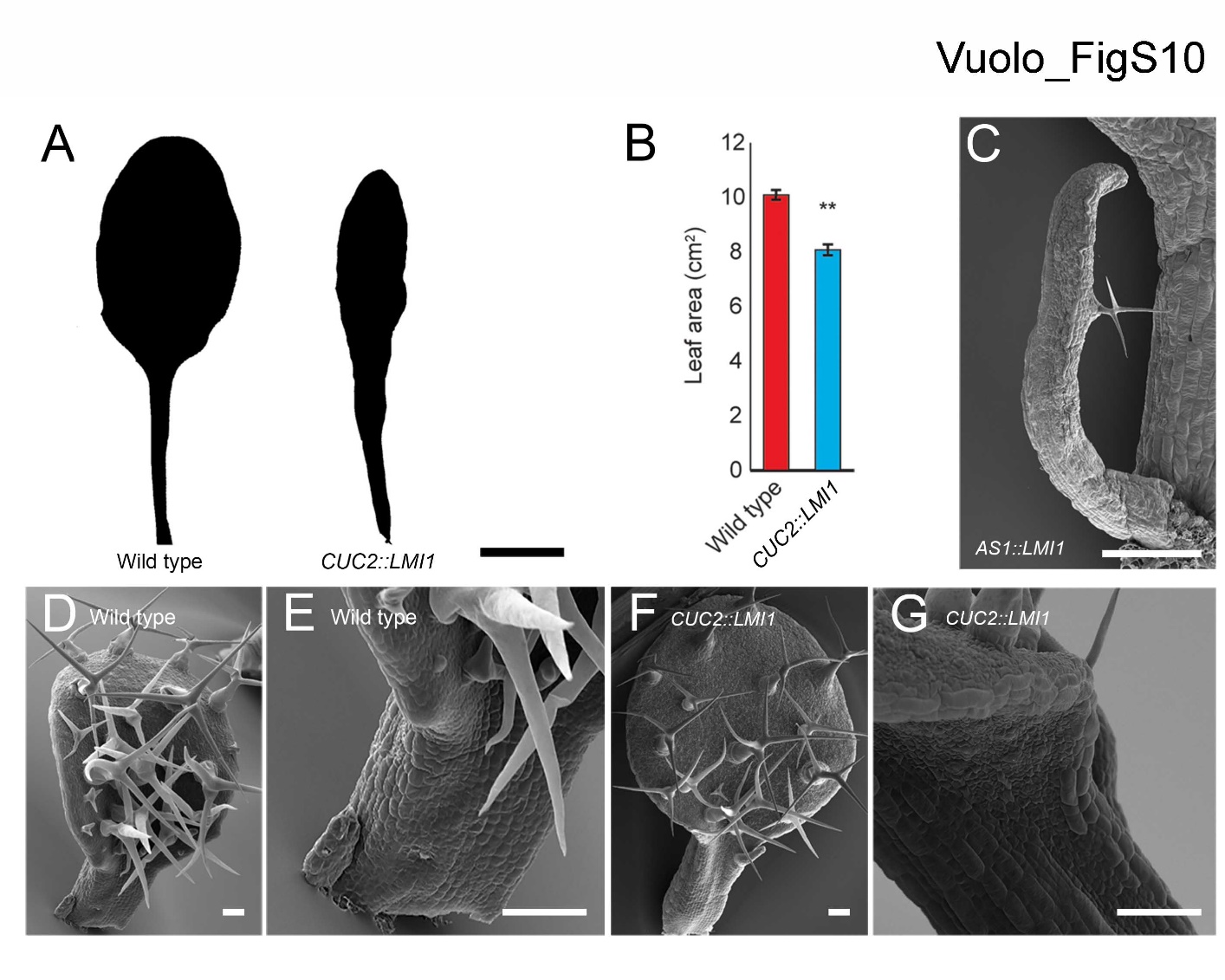


**Supplemental Figure 10. *LMI1* expression in the proximal leaf domain reduces leaf size.  (A)** Leaf 8 silhouettes of wild type (left) and *CUC2::LMI1* (right). **(B)** Leaf area quantification of wild-type (red) and *CUC2::LMI1* (cyan) leaves. Graph shows mean values ± standard deviation; ** indicates *P* < 0.01, Student’s t-test; n > 10. **(C)** Scanning electron micrograph of a leaf in a T1 *ChAS1::LMI1* plant, this phenotype showed ~35% penetrance in the T1 generation. n = 25 **(D-G)** Scanning electron micrographs of (D-E) wild type and (F-G) *CUC2::LMI1.* Scale bar: 50 µm. n ≥10.


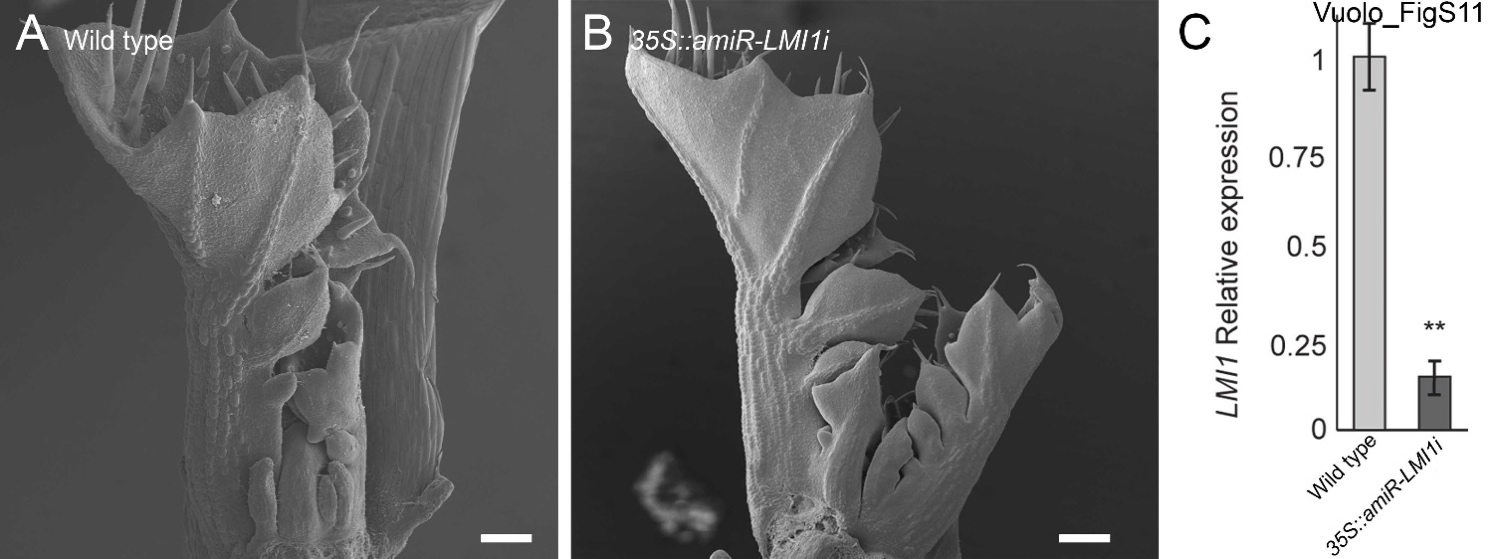


**Supplemental Figure 11. *LMI1* knock-down lines in *C. hirsuta* develop leaves instead of stipules. (A-B)** SEM of leaf 5 in *C. hirsuta* wild type (A) and *35S::amiR-LMI1* (B), representative shown of 10 independent transgenic lines with the same phenotype. Scale bars: 100 μm. Note that blow-ups of these same images are shown in Figure 4E-F, respectively, n ≥10. **(C)** Real time expression plot of *LMI1* transcript levels in wild type (light grey) and *35S::amiR-LMI1i* (dark grey) seedlings. (**) *P* < 0.01. n = 3.


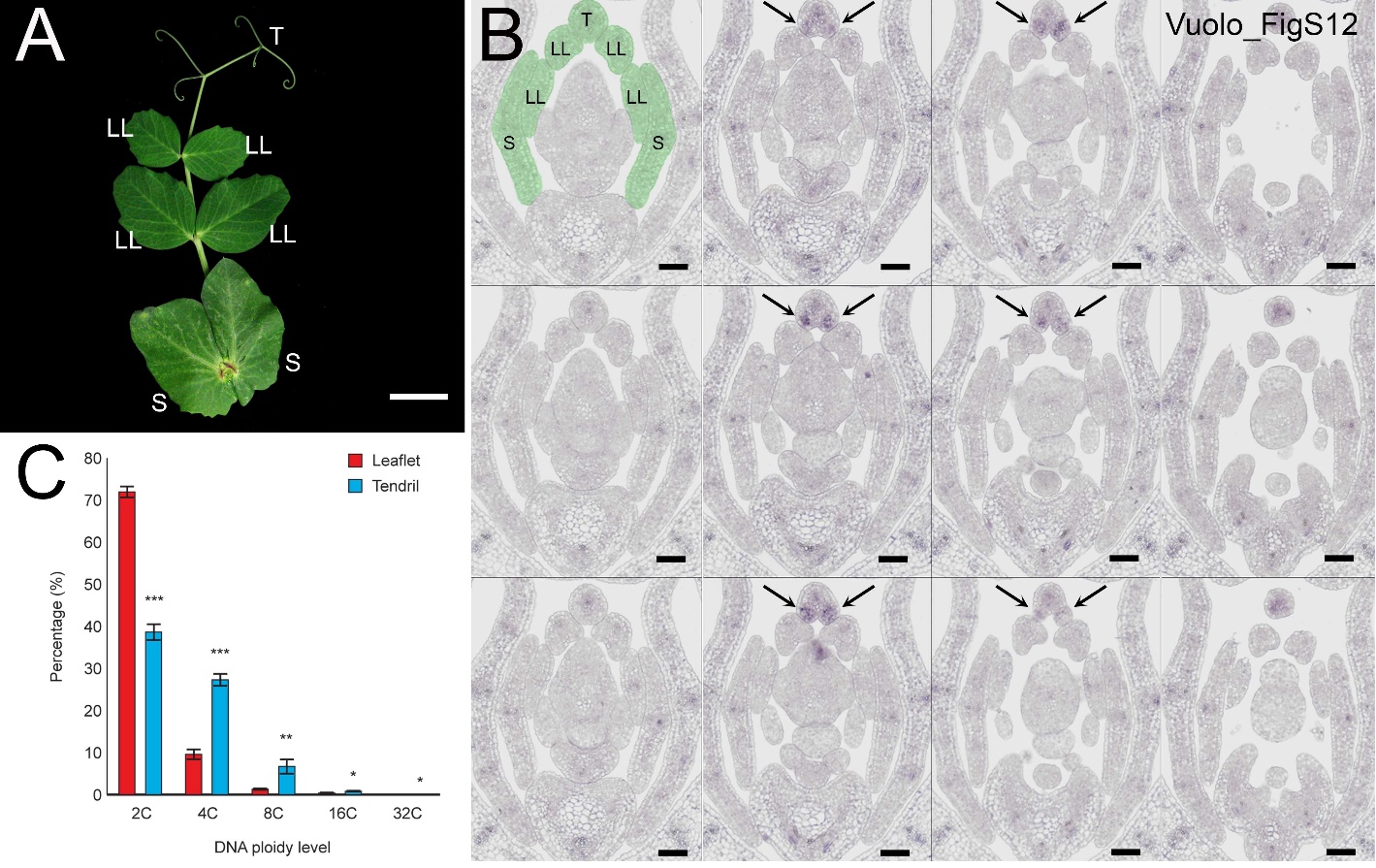


**Supplemental Figure 12. The *LMI1* orthologue *Tendril-less* is expressed in tendrils and leaflets, but not in stipules of *Pisum sativum* leaves. (A)** *Pisum sativum* wild-type leaf 5 showing the wide, leaf-like stipules (S) in the proximal domain of the leaf, the lateral leaflets (LL) in the middle, and the thin tendrils (T) at the distal portion of the leaf. **(B)**  *in situ* hybridization of *Tendril-less* mRNA on serial transverse sections of wild-type *P. sativum* leaves. In (B) note strong expression (black arrows) in tendrils, while no expression is observed in stipules. In the first top-left panel, different parts of a single leaf shaded green and labeled as in (A). Scale bars: 1 cm (A), 50 µm (B). n ≥5. **(C)** Ploidy levels quantification at the flow cytometer of *P. sativum* leaflet (red) and tendril (blue) nuclei. n=5, mean % ± sem, ANOVA **: P<0.01, ***: P<0.001

**Supplemental Table S1 – Phenotyping of multiple mutants in *lmi1* background**

This table reports the number of observations made during the phenotype analysis of the multiple mutants described in Figure 1G and Supplemental Figure S1K. Different alleles of *lmi1* loss of function mutants were previously shown to have an ectopic basal lobe in the adult leaves (Saddic et al. 2006). Here we document the number of plants showing at least one lobed versus not lobed leaf phenotype from the 10^th^ leaf onwards for various genotypes. N=25

| Genotype (n=25) Lobed leaf plants Not lobed leaf plants | | |
| --- | --- | --- |
| Col-0 / *cuc2-3 / wox3-2 / knat2-5;knat6-2;bp-9 / bp-9 / stm-2* | 0 | 25 |
| *lmi1-2* | 25 | 0 |
| *knat2-5;knat6-2;bp-9;lmi1-2* | 25 | 0 |
| *cuc2-3;lmi1-2* | 25 | 0 |
| *wox3-2;lmi1-2* | 0 | 25 |
| *stm-2;lmi1-2* | 25 | 0 |
| *bp-9;lmi1-2* | 25 | 0 |

**Supplemental Table S2 – Model Parameters table**

Parameters for cell-population models. Unless otherwise noted, all simulations use the parameter values specified for Fig. 4B (third column).

| Parameter | |  | Simulation | | |
| --- | --- | --- | --- | --- | --- |
| Name | Symbol |  | Fig. 4B | Fig. S8C | Fig. S8D |
| Initial population size |  |  | 1 |  |  |
| Rate of s-phase |  |  | 8 |  | [0,20] |
| Rate of transition to endoreduplication |  |  | 10 | [0,20] |  |
| Differentiation time | *T_diff_* |  | 1 |  |  |
| Proliferation end time | *T_prolif_* |  | 0.5 |  |  |
| Endoreduplication window | [*T_endo0_,T_endo1_*] |  |  |  |  |
| Maximum ploidy |  |  |  |  |  |
| Time-step |  |  | 0.00325 |  |  |
| Endoreduplication and proliferation always have the same duration (i.e. *T_prolif_ =T_endo1_ –T_endo0_*). | | | | | |
| In all simulations, the population size for cells with a ploidy exceeding was negligable and thus ignored. | | | | | |

**Supplemental Table S3 - Primers used in this study**

| **Name** | **Experiment** | **Sequence** |
| --- | --- | --- |
| knat2-5fw | Genotyping KNAT2 | CGCTTCTCATCCTTTGTATC |
| knat2-5rv | Genotyping KNAT2 | TACCCATCAGTCTCTTAATG |
| knat6-2fw | Genotyping KNAT6 | ACAATTTCCATTCGGCCGGTGATT |
| knat6-2rv | Genotyping KNAT6 | GAAGATAAACCCTAGCTACAAG |
| bp-9 mut | Genotyping BP | CTTATTTCAGTAAGAGTGTGGGGTTTTGG |
| bp-9 fw wt | Genotyping BP | TGTTAAGGGTTAGAACACCATG |
| bp-9 rv wt | Genotyping BP | GACAACAGCACCACTCCTCAAA |
| wox3-2 wt fw | Genotyping WOX3 | CTAAGTGTTTGGAGATAGCATCAC |
| wox3-2 wt rv | Genotyping WOX3 | ACATGGGAGAAGGATGAGAGCAGC |
| wox3-2 mut primer | Genotyping WOX3 | AGCTGTTGCCCGTCTCACTGGTG |
| lmi1-2 fw | Genotyping LMI1 | AATTTCTTGGGATCCAAATGC |
| lmi1-2 rv | Genotyping LMI1 | CTCAATCTCGCATCCAAGAAC |
| Lba-1 | Genotyping LMI1 | TGGTTCACGTAGTGGGCCATCG |
| CYCD4;2 fw qRT | qRT CYCD4;2 | TCAGAATCCAAGCCCTTGGT |
| CYCD4;2 rv qRT | qRT CYCD4;2 | ACGGTGTCACTGCCCGTAAC |
| WEE1 fw qRT | qRT WEE1 | TGGTGCTGGACATTTCAGTCGG |
| WEE1 rv qRT | qRT WEE1 | GGATATTACTCCTCGTGGTTTGAAAATG |
| WEE1-1st-Rv | ChIP-qRT WEE1 1^st^ BS | ATTGGTTCCTTGGATTCCGAG |
| WEE1-1st-Fw | ChIP-qRT WEE1 1^st^ BS | ATATTAAACCGAAATATACCGCGG |
| WEE1-2nd-Rv | ChIP-qRT WEE1 2^nd^ BS | TATTACTTTGGGTGGTGATTCAAC |
| WEE1-2nd-Fw | ChIP-qRT WEE1 2^nd^ BS | CTAAAAGACGAGAGCGCGC |
| LMI1-qRT-Fw | LMI1 qRT | AATCTCTTCCGGGACCACC |
| LMI1-qRT-Rv | LMI1 qRT | CCTCCTGTGATTTGATTGGTG |
| GAPDH F | GAPDH qRT | TGACCACCGTCCACTCCATCAC |
| GAPDH R | GAPDH qRT | GCTCTTCCACCTCTCCAGTCCTTC |
| SalI_AtWEE1p_F | WEE1p amplification | acgcGTCGACgttttatatcccacattttag |
| XmaI_AtWEE1p-R | WEE1p amplification | tcccCCCGGgacgatgaatattattactttg |
